# How do Japanese rate the severity of different diseases and injuries?—an assessment of disability weights for 231 health states by 37,318 Japanese respondents

**DOI:** 10.1186/s12963-021-00253-4

**Published:** 2021-04-23

**Authors:** Shuhei Nomura, Yoshiko Yamamoto, Daisuke Yoneoka, Juanita A. Haagsma, Joshua A. Salomon, Peter Ueda, Rintaro Mori, Damian Santomauro, Theo Vos, Kenji Shibuya

**Affiliations:** 1 Department of Health Policy and Management, School of Medicine, Keio University, Tokyo, Japan; 2 Department of Global Health Policy, Graduate School of Medicine, The University of Tokyo, Tokyo, Japan; 3 National Center for Child Health and Development, Tokyo, Japan; 4 Division of Biostatistics and Bioinformatics, Graduate School of Public Health, St. Luke’s International University, Tokyo, Japan; 5 Department of Public Health, Erasmus MC University Medical Center Rotterdam, Rotterdam, Netherlands; 6 Center for Primary Care and Outcomes Research, Stanford University School of Medicine, California, USA; 7 Clinical Epidemiology Division, Department of Medicine, Solna, Karolinska Institutet, Stockholm, Sweden; 8 Graduate School of Medicine, Kyoto University, Kyoto, Japan; 9 School of Public Health, The University of Queensland, Queensland, Australia; 10 Queensland Centre for Mental Health Research, Queensland, Australia; 11 Institute for Health Metrics and Evaluation, University of Washington, Seattle, USA; 12 Institute for Population Health, King’s College London, London, UK

**Keywords:** Japan, Disability weight, Disease burden, Disability-adjusted life years

## Abstract

**Background:**

Disability weights (DWs) are weight factors that reflect the severity of health states for estimates of disability-adjusted life years. A new set of global DWs was published for the Global Burden of Diseases and Injuries (GBD) 2013 study, which relied on sampling from various world regions, but included little data for countries in East Asia. This study aimed to measure DWs in Japan using comparable methods, and compare the results with previous estimates from the GBD 2013 DW study.

**Methods:**

We conducted a web-based survey in 2019 to estimate DWs for 231 health states for the Japanese population. The survey included five new health states but otherwise followed the method of the GBD DW measurement study. The survey consisted of 15 paired comparison (PC) questions and 3 population health equivalence questions (PHE) per respondent. We analyzed PC data using probit regression and rescaled results to DW units between 0 (equivalent to full health) and 1 (equivalent to death).

**Findings:**

We considered 37,318 nationally representative respondents. The values of the resulting DWs ranged from 0.707 (95% uncertainty interval (UI) 0.527–0.842) for spinal cord injury at neck level (untreated) to 0.004 (UI 0.001–0.009) for mild anemia. High correlation between Japanese DW and GBD 2013 DW was observed, but there was considerable disagreement. Out of 226 comparable health states, 55 (24.3%) showed more than a factor-of-two difference, of which 41 (74.6%) had a higher value in Japanese DW. Many of the health states with higher DW in the Japan study were injuries, including amputation and fracture, and hearing and vision loss, while mental, behavioral, and substance use disorders generally tended to be lower.

**Conclusions:**

This study has created an empirical basis for assessment of Japanese DWs of health status. The findings from this study based on the Japanese population suggest that there might be contextual differences in rating the severity of health states compared to previous surveys conducted elsewhere.

**Supplementary Information:**

The online version contains supplementary material available at 10.1186/s12963-021-00253-4.

## Background

The Global Burden of Disease (GBD) study is a global collaborative project since the 1990s to evaluate the contribution of diseases, injuries, and risks on population health in the world [1]. GBD study summarizes health loss in disability-adjusted life years (DALYs) which are the sum of years of life lost (YLLs) and years lived with disability (YLDs) [2]. As a general concept, YLL reflects the burden of premature mortality from diseases and is calculated by multiplying the number of deaths and standard life expectancy at age of death and YLDs reflects the burden of morbidity and is calculated by multiplying the number of prevalent cases of disease by a disability weight (DW) that reflects the severity of the disabling consequences of disease. A major advantage of the DALY is that it indicates not only the burden of mortality and morbidity separately but also integrated in one number that enables to compare disease burden across all diseases.

DWs are weight factors that reflect the severity of health states. In the GBD 2010 DW study, the methodology to assess DWs had been revised considerably to incorporate the views of the general public rather than relying on the opinion of a select group of global public health experts who provided health state valuations for earlier rounds of GBD. Face to face and telephone surveys were conducted in Peru, Indonesia, Bangladesh, Tanzania, and the USA, supplemented by an open access web-based survey [3]. Instead of person-trade off methods used previously, the surveys were based on paired comparison (PC) questions eliciting valuations based on asking “who is the healthier?” between two persons, each described with a short description in lay terms of the main aspects of their health state [3, 4]. DWs for a parsimonious set of 220 health states covering all disabling outcomes of the diseases and injuries quantified in GBD were derived. Following these initial surveys, there was criticism of the wording of some of the health state descriptions. When the opportunity of a new DW study using a web-based survey in four European countries arose, some lay descriptions were altered to include key components of disability such as the effect of social isolation in someone with more severe hearing loss and incontinence as part of the description of spinal cord injury [5]. The modifications of lay descriptions resulted in a change of DWs in the expected direction.

The GBD 2010 DW study and the subsequent European surveys showed a high level of consistency of responses between countries and educational attainment [3]. However, these studies included few respondents from East Asian countries where cultural differences may influence health state valuations more than has been found elsewhere. Several previous studies suggest that the DWs in East Asian countries may differ from that of Western countries [6, 7]. Rigorous evaluation of the potential for contextual differences to rate the severity of health states in different settings is important for the further development of disease burden studies. In this study, we aimed to estimate DWs in the Japan—an East Asian country that has a unique healthcare system in that social health insurance offers universal health care [8–10]—using the same methodology as the previous GBD DW studies. The estimated Japanese DWs were compared to the estimates that have been used in GBD since 2013. We added health states that are common in the Japanese population and that were not included in previous DW studies.

## Methods

For the assessment of the DWs for Japan, we followed the same procedure as was used in the previous DW measurement studies in a web-based survey design [3, 5, 11].

### Lay description of health states

DWs for a set of 231 health states were assessed. The health states consisted of the following categories: 166 health states that were included in the GBD 2010 DW study and repeated in unaltered form in the European study (GBD 2010 original); 33 health states for which the lay descriptions were revised for the European DW study (GBD 2010 modified); 27 health states that were included only in the European DW study (European original); and 5 new health states. The five new health states included two generic drug health states (drug dependence and mild drug dependence) rather than the drug-specific ones (i.e., opioid, cannabis, amphetamine, and cocaine) that the previous studies had, one existing health state for which the lay description was expanded (vaginal discharge), and two completely new health states, cancer-post treatment and dermatitis. We excluded health states of the GBD 2010 and 2013 studies that were not relevant or rare in the Japanese context such as lymphatic filariasis, fetal alcohol syndrome, lower airway burns, and kwashiorkor. The new Japanese health state for dermatitis replaced the three GBD health states for disfigurement with itch or pain. The list of the 231 health states and their origins (GBD 2020 original, etc.) is presented in Additional file 1: table 1.

By using professional outsourced translation services, the lay descriptions were translated from English to Japanese and back-translated from Japanese to English and the consistency of meaning was verified by independent clinical experts from the authors’ institutions. The framing of the pair wise comparison questions was varied between chronic (“imagine each of the conditions in the pair would last for a person’s life time”) and temporary (“imagine each of the conditions in the pair would last for one week”). Of the 231 health states, 34 were framed as chronic only, 106 as temporary only, and 91 as either chronic or temporary. The list of lay descriptions of the 231 health states and their designation as chronic, temporary or both is also presented in Additional file 1: table 1.

### Study population

The participants of the web-based survey were those registered to the panel of web survey company (Cross Marketing Inc.) [12]. The panel included those aged from 18 to 70 years old. Membership of the panel is on a voluntary basis, and the incentives to join the panel are that those who respond to questionnaires administered by the company are provided with “points” based on the survey volume. Points can be used to purchase products and services from partner companies [12].

In this study, the target number of study participants was set at approximately 40,000, and in order to ensure national representation, a quota sampling method based on age, gender, and prefecture population ratios obtained from the 2015 National Census was used to finally set 37,318 participants as the fixed number. Participation was first-come-first-served and the survey was closed when the number of respondents reached the pre-determined target population by age, gender, and prefecture. The survey began on 25 January 2019, and the target was reached on 30 January 2019.

The respondents were required to respond to each question so that there was no missing value. The respondents had given consent to the terms and conditions and privacy policy that the company sent with the invitation of questionnaires detailing how the company deals with confidential information of individuals. This sample size was determined based on statistical considerations as well as sample sizes used in similar research [5]. Characteristics of respondents are shown in Table 1 in comparison with the whole population distribution in Japan, derived from the National Census 2010 and 2015 [13, 14]. Except for educational level, most demographic characteristics were similar to the distribution of the whole population. The percentage of university graduates among respondents (46.2%) was larger than the whole population (16.1%).

**Table 1:** Background of respondents

	Respondents		National population [14]
Variable	Number	Percentage	Percentage
Gender			
Male	19,462	52.2	48.7
Female	17,856	47.9	51.3
Age^a^			
–19	756	2.0	2.5
20–29	5102	13.7	12.9
30–39	6481	17.4	16.2
40–49	7642	20.5	19.1
50–59	6417	17.2	16.0
60–69	6995	18.7	18.8
70–	3925	10.5	14.5
Prefecture			
Hokkaido	1598	4.3	4.2
Aomori	350	0.9	1.0
Iwate	338	0.9	1.0
Miyagi	801	2.2	1.8
Akita	288	0.8	0.8
Yamagata	267	0.7	0.9
Fukushima	428	1.2	1.5
Ibaraki	667	1.8	2.3
Tochigi	386	1.0	1.6
Gunma	357	1.0	1.6
Saitama	2095	5.6	5.7
Chiba	1809	4.9	4.9
Tokyo	5013	13.4	10.6
Kanagawa	3061	8.2	7.2
Niigata	618	1.7	1.8
Toyama	281	0.8	0.8
Ishikawa	332	0.9	0.9
Fukui	188	0.5	0.6
Yamanashi	169	0.5	0.7
Nagano	537	1.4	1.7
Gifu	561	1.5	1.6
Shizuoka	977	2.6	2.9
Aichi	2432	6.5	5.9
Mie	414	1.1	1.4
Shiga	345	0.9	1.1
Kyoto	815	2.2	2.1
Osaka	2827	7.6	7.0
Hyogo	1661	4.5	4.4
Nara	470	1.3	1.1
Wakayama	227	0.6	0.8
Tottori	142	0.4	0.5
Shimane	149	0.4	0.5
Okayama	592	1.6	1.5
Hiroshima	871	2.3	2.2
Yamaguchi	304	0.8	1.1
Tokushima	212	0.6	0.6
Kagawa	276	0.7	0.8
Ehime	388	1.0	1.1
Kochi	153	0.4	0.6
Fukuoka	1872	5.0	4.0
Saga	193	0.5	0.7
Nagasaki	376	1.0	1.1
Kumamoto	399	1.0	1.4
Oita	249	0.7	0.9
Miyazaki	221	0.6	0.9
Kagoshima	318	0.9	1.3
Okinawa	291	0.8	1.1
Highest educational level [13]			
High school	13,591	36.4	37.5
University^b^	17,223	46.2	16.1
Marital status			
Married	21,394	57.3	58.5
Divorced	2666	7.1	5.2
Widowed	865	2.3	8.9
Never married	12,258	32.9	27.3

^a^Denominator of national population was the number of population aged 18 and above

^b^Includes graduate schools

### Web survey

We used the same questionnaire as the GBD 2010 and European DW study that consisted of three parts. The first part consisted of questions about sociodemographic and geographic characteristics of participants, the second part consisted of PC questions, and the last part consisted of population health equivalence (PHE) questions. In the PC part, each participant answered 15 questions with either chronic or temporary framing for computer-generated random selection of health states pairs. Participants were allocated to the chronic or temporary framing according to the ratio of the number of health states in each set, which was 125 or 197. In the PHE part, each participant answered three questions comparing two randomly selected health programs: one prevented 1000 people to die immediately and another prevented randomly selected number of people such as 1500, 2000, 3000, 5000, and 10,000 to suffer from one of the selected 28 health states for the rest of their lives. Respondents were instructed to choose which program had produced the greatest amount of health gain.

### Data analysis

All analyses were performed with STATA (version 15) and R (version 3.6.1). The PC data were plotted with a heat map that represents the probability of selecting the first health state in the pair as the healthier of the two states. We tested reliability of the PC responses by deliberately repeating the first pair in the last PC question, a similar test-retest procedure to that of the European DW study [5].

A probit regression model was used to estimate the latent preference of the health states using the PC data. The response variable was given a value of 1 if the first health state in the pair was selected as the healthier and 0, otherwise. The regression included indicator variables for each health state, which took the value of 1 if the state was the first one presented in the pair, −1 if it was the second state in the pair, −1 if the state was part of the PC, and 0 otherwise. A linear regression model was used to anchor the estimated results of the probit regression model, which were logit transformed to map onto a DW scale ranging from 0 to 1, based on the PHE responses. Then, Monte Carlo integration using normal random samples was used to estimate the mean of DWs [15]. Lastly, 1000 bootstrap iterations were implemented to compute 95% uncertainty intervals (UIs).

We also compared the estimated Japanese DWs for 226 health states (excluding 5 new states for the present study) with the GBD 2013 DWs to assess the health state or health category differences in the DWs.

In addition, regression analysis was performed to assess what symptoms mentioned in the lay descriptions were associated with the difference between the Japanese DW and the GBD DW. We identified eleven symptom categories based on the wording of the lay descriptions (Additional file 1: table 1), including mobility, pain, mental symptoms, fatigue, disfigurement, sensory symptoms, infection/diarrhea, substance use, activities of daily living (ADL), cognitive symptoms, and other physical symptoms. We constructed a linear regression model with outcomes of proportional differences between 226 Japanese and GBD 2013 DWs (excluding 5 states that were not included in the GBD 2013 study). All eleven symptom categories were simultaneously entered into the models.

### Role of the funding source

The funders of the study had no role in the study design, data collection, data analysis, data interpretation, or writing of the paper. The authors had full access to all the data in the study and had final responsibility to submit for publication.

## Results

### Paired comparison

**Fig. 1:**
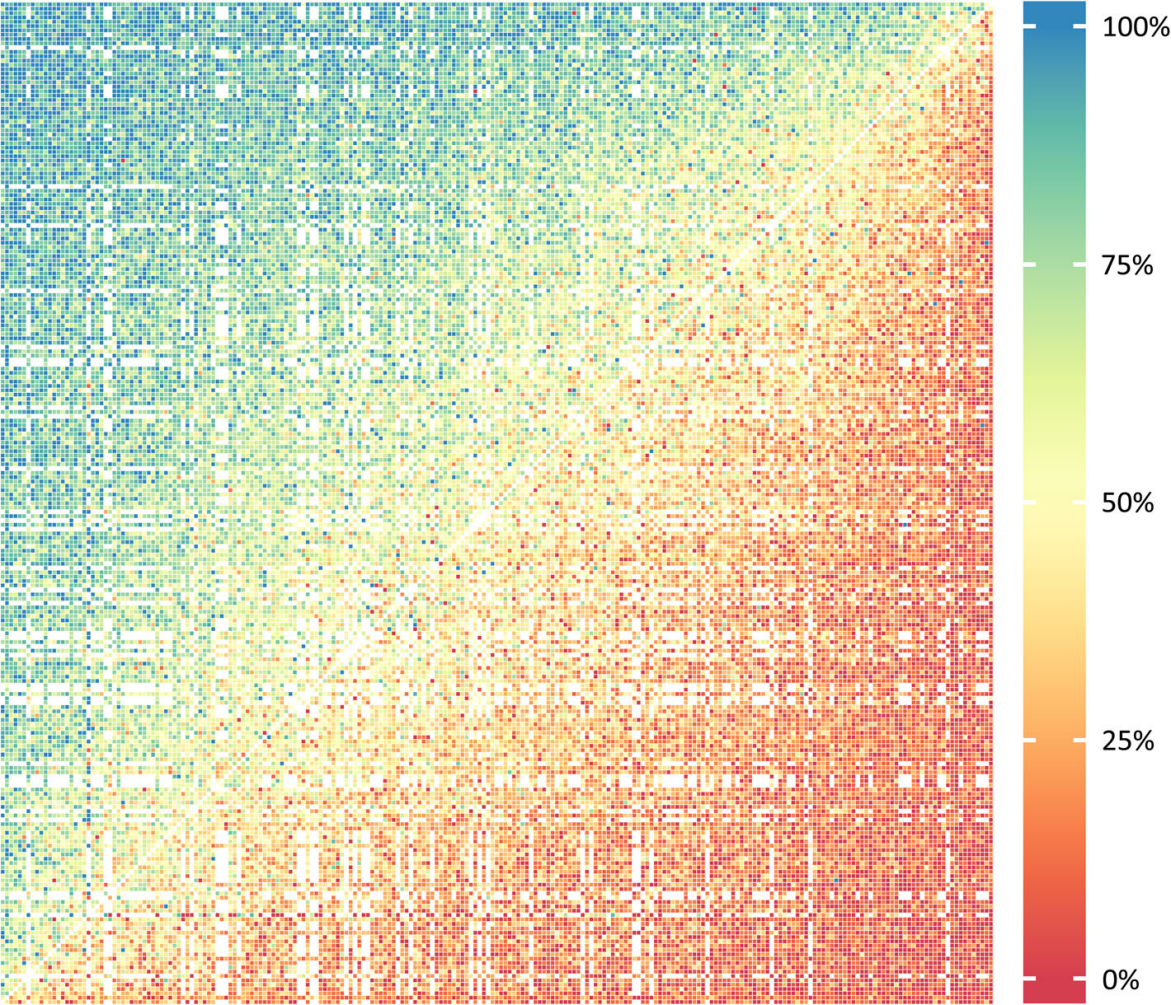
Response probabilities for paired comparison. Red represents a probability of less than 0.25. Blue represents a probability greater than 0.75. Green, yellow, and orange correspond to probabilities between 0.25 and 0.75. A smooth transition of colors between the upper left and lower right corners exhibits indicates low measurement error and good internal consistency, while a completely random combination of colors reflects high measurement error and poor internal consistency. Note that not all possible 231 × 231 pairs were evaluated by pairwise comparison, which is indicated by some blanks in the figure

The heat map of the responses of the PC is shown in Fig. 1. Each cell in the heat map indicates the response probability for one pair of health states. The colors of the heat map correspond to the probability that the first health state in a pair comparison is chosen as the healthier outcome. The colors in the heat map show a smooth transition of preferences in each comparison, indicating high internal consistency. A reliability check of PC responses showed that inconsistent responses to the same pair occurred in 21.6% of cases.

### Population health equivalence

**Fig. 2:**
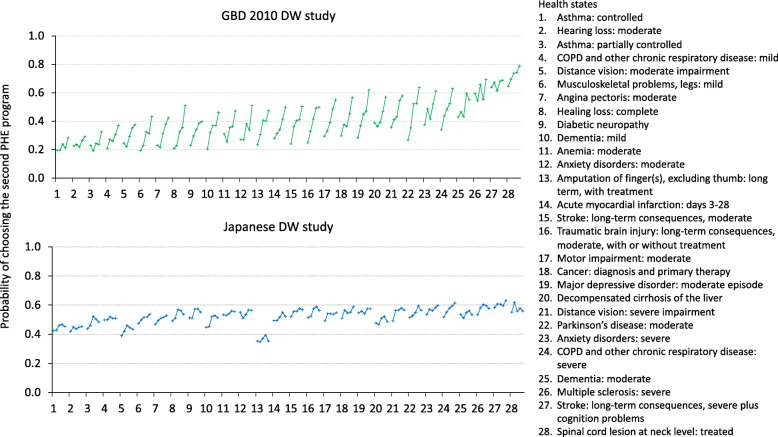
Probability of choosing the second program for each of the 28 health states that were evaluated with the population health equivalence (PHE) questions, in the present study (bottom panel) compared to results in the GBD 2010 study (top panel). GBD, Global Burden of Disease study; DW, disability weight. Each line represents one health state and each dot represents a bid within one health state

Figure 2 shows the proportion of participants that selected the health programs treating health states with randomly assigned a bid of the number of people to the program treating 1000 people of disease of immediate death. We expected that the proportion of choosing the second health program increased with increasing bid; however, the correlation between the proportion of choosing the second program and bid was low (0.32 for Spearman’s correlation coefficient). In addition, the probability of choosing the second program converged around 50%, regardless of the severity of health states, in contrast to the PHE responses from the GBD study that had an increasing trend in probabilities with the severity of health states (results also shown in Fig. 2).

### Estimates of Japanese disability weights

Because of the evident lack of discernment in the PHE response in our study, we used the PHE data from the GBD 2010 DW study to anchor our regression estimates from the PC onto DW scale ranging from 0 to 1. Estimated Japanese DW for the 231 health states, in comparison with the GBD 2013 study are shown in Table 2. The highest DW was 0.707 (95% UI 0.527–0.842) for spinal cord injury at neck level (untreated), followed by 0.675 (0.506–0.822) of intensive care unit admission and 0.653 (0.483–0.798) of multiple sclerosis, severe. The lowest DW was 0.004 (0.001–0.009) of mild anemia, followed by 0.005 (0.002–0.012) of mild distance vision loss, and 0.006 (0.003–0.013) of controlled asthma.

**Table 2:** Estimated Japanese disability weights (95% uncertainty interval), compared to the GBD 2013 disability weights

Id	Health state	Japanese DW	GBD 2013 DW [11]	Factor of two or greater difference	Factor of three or greater difference
Infectious disease					
1	Infectious disease, acute episode, mild	0.012 (0.005–0.022)	0.006 (0.002–0.012)		
2	Infectious disease, acute episode, moderate	0.424 (0.289–0.577)	0.051 (0.032–0.074)	Japan > GBD	Japan > GBD
3	Infectious disease, acute episode, severe	0.242 (0.163–0.340)	0.133 (0.088–0.190)		
4	Infectious disease, post-acute consequences (fatigue, emotional lability, insomnia)	0.074 (0.047–0.106)	0.219 (0.148–0.308)	Japan < GBD	
5	Diarrhea, mild	0.119 (0.079–0.165)	0.074 (0.049–0.104)		
6	Diarrhea, moderate	0.250 (0.170–0.345)	0.188 (0.125–0.264)		
7	Diarrhea, severe	0.387 (0.263–0.517)	0.247 (0.164–0.348)		
8	Epididymo-orchitis	0.204 (0.139–0.283)	0.128 (0.086–0.180)		
9	Herpes zoster	0.181 (0.123–0.257)	0.058 (0.035–0.090)	Japan > GBD	Japan > GBD
10	HIV cases, symptomatic, pre-AIDS	0.200 (0.140–0.275)	0.274 (0.184–0.377)		
11	HIV/AIDS cases, receiving ARV treatment	0.155 (0.108–0.219)	0.078 (0.052–0.111)		
12	AIDS cases, not receiving ARV treatment	0.394 (0.268–0.527)	0.582 (0.406–0.743)		
13	Ear pain	0.058 (0.037–0.085)	0.013 (0.007–0.024)	Japan > GBD	Japan > GBD
14	Tuberculosis, HIV infected	0.267 (0.181–0.372)	0.408 (0.274–0.549)		
15	Tuberculosis, not HIV infected	0.254 (0.175–0.348)	0.333 (0.224–0.454)		
Cancer					
16	Cancer, diagnosis and primary therapy	0.174 (0.121–0.243)	0.288 (0.193–0.399)		
17	Cancer, metastatic	0.230 (0.158–0.312)	0.451 (0.307–0.600)		
18	Cancer, after treatment	0.079 (0.052–0.115)	NA		
19	Mastectomy	0.075 (0.047–0.108)	0.036 (0.020–0.057)	Japan > GBD	
20	Stoma	0.146 (0.099–0.200)	0.095 (0.063–0.131)		
21	Terminal phase, with medication (for cancers, end-stage kidney/liver disease)	0.589 (0.425–0.743)	0.540 (0.377–0.687)		
22	Terminal phase, without medication (for cancers, end-stage kidney/liver disease)	0.537 (0.378–0.693)	0.569 (0.389–0.727)		
Cardiovascular and circulatory disease					
23	Acute myocardial infarction: days 1-2	0.253 (0.173–0.353)	0.432 (0.288–0.579)		
24	Acute myocardial infarction, days 3-28	0.032 (0.019–0.050)	0.074 (0.049–0.105)	Japan < GBD	
25	Angina pectoris, mild	0.019 (0.010–0.033)	0.033 (0.020–0.052)		
26	Angina pectoris, moderate	0.040 (0.024–0.062)	0.080 (0.052–0.133)		
27	Angina pectoris, severe	0.163 (0.111–0.227)	0.167 (0.110–0.240)		
28	Cardiac conduction disorders and cardiac dysrhythmias	0.426 (0.297–0.561)	0.224 (0.151–0.312)		
29	Claudication	0.020 (0.011–0.034)	0.014 (0.007–0.025)		
30	Heart failure, mild	0.016 (0.008–0.028)	0.041 (0.026–0.062)	Japan < GBD	
31	Heart failure, moderate	0.041 (0.025–0.061)	0.072 (0.047–0.103)		
32	Heart failure, severe	0.240 (0.166–0.336)	0.179 (0.122–0.251)		
33	Stroke: long-term consequences, mild	0.019 (0.010–0.033)	0.019 (0.010–0.032)		
34	Stroke: long-term consequences, moderate	0.044 (0.027–0.067)	0.070 (0.046–0.099)		
35	Stroke: long-term consequences, moderate plus cognition problems	0.108 (0.074–0.150)	0.316 (0.206–0.437)	Japan < GBD	
36	Stroke: long-term consequences, severe	0.550 (0.386–0.701)	0.552 (0.377–0.707)		
37	Stroke: long-term consequences, severe plus cognition problems	0.579 (0.411–0.732)	0.588 (0.411–0.744)		
Diabetes and digestive and genitourinary disease					
38	Diabetic foot	0.064 (0.041–0.093)	0.020 (0.010–0.034)	Japan > GBD	Japan > GBD
39	Diabetic neuropathy	0.098 (0.063–0.136)	0.133 (0.089–0.187)		
40	Chronic kidney disease (stage IV)	0.106 (0.070–0.151)	0.104 (0.070–0.147)		
41	End-stage renal disease, on dialysis	0.278 (0.189–0.382)	0.571 (0.398–0.725)	Japan < GBD	
42	End-stage renal disease, with kidney transplant	0.018 (0.010–0.032)	0.024 (0.014–0.039)		
43	Decompensated cirrhosis of the liver	0.101 (0.067–0.144)	0.178 (0.123–0.250)		
44	Gastric bleeding	0.541 (0.387–0.690)	0.325 (0.209–0.462)		
45	Crohn’s disease or ulcerative colitis	0.219 (0.152–0.308)	0.231 (0.156–0.320)		
46	Benign prostatic hypertrophy: symptomatic	0.096 (0.064–0.136)	0.067 (0.043–0.097)		
47	Urinary incontinence	0.210 (0.143–0.292)	0.139 (0.094–0.198)		
48	Stress incontinence	0.014 (0.007–0.026)	0.020 (0.011–0.035)		
49	Impotence	0.017 (0.009–0.030)	0.017 (0.009–0.030)		
50	Infertility, primary	0.009 (0.004–0.018)	0.008 (0.003–0.015)		
51	Infertility, secondary	0.008 (0.003–0.016)	0.005 (0.002–0.011)		
Chronic respiratory disease					
52	Asthma, controlled	0.006 (0.003–0.013)	0.015 (0.007–0.026)	Japan < GBD	
53	Asthma, partially controlled	0.044 (0.027–0.065)	0.036 (0.022–0.055)		
54	Asthma: uncontrolled	0.212 (0.145–0.294)	0.133 (0.086–0.192)		
55	COPD and other chronic respiratory problems, mild	0.008 (0.003–0.016)	0.019 (0.007–0.026)	Japan < GBD	
56	COPD and other chronic respiratory problems, moderate	0.232 (0.158–0.319)	0.225 (0.153–0.310)		
57	COPD and other chronic respiratory problems, severe	0.299 (0.203–0.405)	0.408 (0.273–0.556)		
Neurological disorders					
58	Dementia, mild	0.037 (0.022–0.056)	0.069 (0.046–0.099)		
59	Dementia, moderate	0.382 (0.263–0.519)	0.377 (0.252–0.508)		
60	Dementia, severe	0.511 (0.358–0.657)	0.449 (0.304–0.595)		
61	Headache: migraine	0.518 (0.365–0.668)	0.441 (0.294–0.588)		
62	Headache: tension-type	0.109 (0.074–0.153)	0.037 (0.022–0.057)	Japan > GBD	
63	Headache: medication overuse	0.185 (0.129–0.262)	0.223 (0.146–0.313)		
64	Multiple sclerosis, mild	0.235 (0.160–0.324)	0.183 (0.124–0.253)		
65	Multiple sclerosis, moderate	0.467 (0.325–0.615)	0.463 (0.313–0.613)		
66	Multiple sclerosis, severe	0.653 (0.483–0.798)	0.719 (0.534–0.858)		
67	Epilepsy, seizures ≥ once a month	0.533 (0.373–0.686)	0.552 (0.375–0.710)		
68	Epilepsy, seizures < once a month	0.396 (0.273–0.525)	0.263 (0.173–0.367)		
69	Parkinson’s disease, mild	0.016 (0.008–0.028)	0.010 (0.005–0.019)		
70	Parkinson’s disease, moderate	0.193 (0.133–0.269)	0.267 (0.181–0.372)		
71	Parkinson’s disease, severe	0.527 (0.372–0.681)	0.575 (0.396–0.730)		
Mental, behavioral, and substance use disorders					
72	Alcohol use disorder, very mild	0.064 (0.040–0.092)	0.123 (0.082–0.177)		
73	Alcohol use disorder, mild	0.219 (0.151–0.306)	0.235 (0.160–0.327)		
74	Alcohol use disorder, moderate	0.312 (0.213–0.422)	0.373 (0.248–0.508)		
75	Alcohol use disorder, severe	0.413 (0.282–0.551)	0.570 (0.396–0.732)		
76	Drug dependence, mild	0.330 (0.229–0.446)	NA		
77	Drug dependence	0.581 (0.419–0.740)	NA		
78	Anxiety disorders, mild	0.014 (0.007–0.026)	0.030 (0.018–0.046)	Japan < GBD	
79	Anxiety disorders, moderate	0.108 (0.072–0.150)	0.133 (0.091–0.186)		
80	Anxiety disorders, severe	0.376 (0.258–0.509)	0.523 (0.362–0.677)		
81	Major depressive disorder, mild episode	0.060 (0.038–0.088)	0.145 (0.099–0.209)	Japan < GBD	
82	Major depressive disorder, moderate episode	0.302 (0.204–0.410)	0.396 (0.267–0.531)		
83	Major depressive disorder, severe episode	0.533 (0.378–0.680)	0.658 (0.477–0.807)		
84	Bipolar disorder: manic episode	0.321 (0.220–0.439)	0.492 (0.341–0.646)		
85	Bipolar disorder: residual state	0.031 (0.017–0.048)	0.032 (0.018–0.051)		
86	Schizophrenia: acute state	0.575 (0.408–0.733)	0.778 (0.606–0.900)		
87	Schizophrenia: residual state	0.412 (0.283–0.554)	0.588 (0.411–0.754)		
88	Anorexia nervosa	0.196 (0.137–0.272)	0.224 (0.150–0.312)		
89	Bulimia nervosa	0.280 (0.191–0.378)	0.223 (0.150–0.312)		
90	Attention deficit hyperactivity disorder	0.052 (0.032–0.076)	0.045 (0.028–0.066)		
91	Conduct disorder	0.243 (0.167–0.332)	0.241 (0.159–0.341)		
92	Asperger’s syndrome	0.099 (0.065–0.142)	0.104 (0.071–0.147)		
93	Autism	0.176 (0.122–0.245)	0.262 (0.176–0.365)		
94	Intellectual disability, borderline	0.014 (0.006–0.024)	0.011 (0.005–0.020)		
95	Intellectual disability, mild	0.047 (0.030–0.071)	0.043 (0.026–0.064)		
96	Intellectual disability, moderate	0.074 (0.047–0.108)	0.100 (0.066–0.142)		
97	Intellectual disability, severe	0.122 (0.083–0.172)	0.160 (0.107–0.266)		
98	Intellectual disability, profound	0.230 (0.159–0.317)	0.200 (0.133–0.283)		
Hearing and vision loss					
99	Hearing loss, mild	0.027 (0.015–0.044)	0.010 (0.004–0.019)	Japan > GBD	
100	Hearing loss, moderate	0.038 (0.023–0.057)	0.027 (0.015–0.042)		
101	Hearing loss, severe	0.208 (0.143–0.294)	0.158 (0.105–0.227)		
102	Hearing loss, profound	0.241 (0.167–0.338)	0.204 (0.134–0.288)		
103	Hearing loss, complete	0.300 (0.206–0.409)	0.215 (0.144–0.307)		
104	Hearing loss, mild, with ringing	0.047 (0.029–0.069)	0.021 (0.012–0.036)	Japan > GBD	
105	Hearing loss, moderate, with ringing	0.119 (0.080–0.166)	0.031 (0.019–0.049)	Japan > GBD	Japan > GBD
106	Hearing loss, severe, with ringing	0.280 (0.193–0.386)	0.261 (0.175–0.360)		
107	Hearing loss, profound, with ringing	0.307 (0.214–0.414)	0.277 (0.182–0.387)		
108	Hearing loss, complete, with ringing	0.379 (0.266–0.514)	0.316 (0.212–0.435)		
109	Distance vision, mild impairment	0.005 (0.002–0.012)	0.003 (0.001–0.007)		
110	Distance vision, moderate impairment	0.051 (0.032–0.074)	0.031 (0.019–0.049)		
111	Distance vision, severe impairment	0.378 (0.266–0.514)	0.184 (0.124–0.260)	Japan > GBD	
112	Distance vision blindness	0.427 (0.299–0.570)	0.187 (0.124–0.260)	Japan > GBD	
113	Near vision impairment	0.012 (0.006–0.023)	0.011 (0.005–0.020)		
Musculoskeletal disorders					
114	Low back pain, mild	0.028 (0.016–0.045)	0.020 (0.011–0.035)		
115	Low back pain, moderate	0.069 (0.044–0.100)	0.054 (0.035–0.079)		
116	Back pain, severe, without leg pain	0.190 (0.132–0.263)	0.272 (0.182–0.373)		
117	Back pain, severe, with leg pain	0.276 (0.190–0.381)	0.325 (0.219–0.446)		
118	Back pain, most severe, without leg pain	0.200 (0.141–0.274)	0.367 (0.227–0.523)		
119	Back pain, most severe, with leg pain	0.276 (0.186–0.379)	0.379 (0.236–0.540)		
120	Neck pain, mild	0.039 (0.023–0.058)	0.053 (0.034–0.078)		
121	Neck pain, moderate	0.063 (0.041–0.091)	0.114 (0.075–0.162)		
122	Neck pain, severe	0.169 (0.115–0.236)	0.229 (0.153–0.317)		
123	Neck pain, most severe	0.144 (0.099–0.200)	0.304 (0.202–0.415)	Japan < GBD	
124	Musculoskeletal problems, lower limbs, mild	0.091 (0.060–0.129)	0.023 (0.013–0.037)	Japan > GBD	Japan > GBD
125	Musculoskeletal problems, lower limbs, moderate	0.142 (0.098–0.196)	0.079 (0.054–0.110)		
126	Musculoskeletal problems, lower limbs, severe	0.327 (0.223–0.438)	0.165 (0.112–0.232)		
127	Musculoskeletal problems, upper limbs, mild	0.039 (0.023–0.058)	0.028 (0.017–0.045)		
128	Musculoskeletal problems, upper limbs, moderate	0.223 (0.153–0.307)	0.117 (0.080–0.163)		
129	Musculoskeletal problems, generalized, moderate	0.255 (0.177–0.352)	0.317 (0.216–0.440)		
130	Musculoskeletal problems, generalized, severe	0.420 (0.293–0.560)	0.581 (0.403–0.739)		
131	Grout: Acute	0.322 (0.221–0.436)	0.295 (0.196–0.409)		
Injury					
132	Amputation of finger(s), excluding thumb	0.063 (0.041–0.092)	0.005 (0.002–0.010)	Japan > GBD	Japan > GBD
133	Amputation of thumb (long term)	0.093 (0.061–0.132)	0.011 (0.005–0.021)	Japan > GBD	Japan > GBD
134	Amputation of one upper limb (with treatment)	0.144 (0.096–0.205)	0.039 (0.024–0.059)	Japan > GBD	Japan > GBD
135	Amputation of one upper limb (long term, without treatment)	0.261 (0.182–0.357)	0.118 (0.079–0.167)	Japan > GBD	
136	Amputation of both upper limbs (long term, with treatment)	0.193 (0.132–0.274)	0.123 (0.081–0.176)		
137	Amputation of both upper limbs (long term, without treatment)	0.422 (0.295–0.562)	0.383 (0.251–0.525)		
138	Amputation of toe	0.080 (0.052–0.114)	0.006 (0.002–0.012)	Japan > GBD	Japan > GBD
139	Amputation of one lower limb (long term, with treatment)	0.096 (0.062–0.140)	0.039 (0.023–0.059)	Japan > GBD	
140	Amputation of one lower limb (long term, without treatment)	0.233 (0.162–0.318)	0.173 (0.118–0.240)		
141	Amputation of both lower limbs (long term, with treatment)	0.146 (0.101–0.203)	0.088 (0.057–0.124)		
142	Amputation of both lower limbs (long term, without treatment)	0.525 (0.377–0.681)	0.443 (0.297–0.589)		
143	Burns, <20% total burned surface area or < 10% total burned surface area if head/neck or hands/wrist involved (long term, with or without treatment)	0.018 (0.009–0.031)	0.016 (0.008–0.032)		
144	Burns, ≥20% total burned surface area (short term, with or without treatment)	0.208 (0.140–0.302)	0.314 (0.211–0.441)		
145	Burns, ≥20% total burned surface area or ≥10% total burned surface area if head/neck or hands/wrist involved (long term, with treatment)	0.126 (0.085–0.176)	0.135 (0.092–0.190)		
146	Burns, ≥20% total burned surface area or ≥10% total burned surface area if head/neck or hands/wrist involved (long term, without treatment)	0.396 (0.274–0.534)	0.455 (0.302–0.601)		
147	Crush injury (short or long term, with or without treatment)	0.185 (0.129–0.257)	0.132 (0.089–0.189)		
148	Dislocation of hip (long term, with or without treatment)	0.035 (0.021–0.053)	0.016 (0.008–0.028)	Japan > GBD	
149	Dislocation of knee (long term, with or without treatment)	0.252 (0.174–0.347)	0.113 (0.075–0.160)	Japan > GBD	
150	Dislocation of shoulder (long term, with or without treatment)	0.132 (0.091–0.187)	0.062 (0.041–0.088)	Japan > GBD	
151	Other injuries of muscle and tendon (includes sprains, strains and dislocations other than shoulder, knee, hip)	0.032 (0.018–0.049)	0.008 (0.003–0.015)	Japan > GBD	Japan > GBD
152	Drowning and nonfatal submersion (short or long term, with or without treatment)	0.079 (0.052–0.114)	0.247 (0.164–0.341)	Japan < GBD	Japan < GBD
153	Fracture of clavicle, scapula or humerus (short or long term, with or without treatment)	0.159 (0.106–0.222)	0.035 (0.021–0.053)	Japan > GBD	Japan > GBD
154	Fracture of face bone (short or long term with or without treatment)	0.184 (0.128–0.259)	0.067 (0.044–0.097)	Japan > GBD	
155	Fracture of foot bones (short term, with or without treatment)	0.094 (0.062–0.132)	0.026 (0.015–0.043)	Japan > GBD	Japan > GBD
156	Fracture of foot bones (long term, without treatment)	0.045 (0.027–0.066)	0.026 (0.015–0.042)		
157	Fracture of hand (short term, with or without treatment)	0.041 (0.025–0.062)	0.010 (0.005–0.019)	Japan > GBD	Japan > GBD
158	Fracture of hand (long term, without treatment)	0.037 (0.022–0.058)	0.014 (0.007–0.025)	Japan > GBD	
159	Fracture of neck of femur (short term, with or without treatment)	0.321 (0.218–0.437)	0.258 (0.172–0.356)		
160	Fracture of neck of femur (long term, with treatment)	0.120 (0.079–0.168)	0.058 (0.038–0.084)	Japan > GBD	
161	Fracture of neck of femur (long term, without treatment)	0.375 (0.260–0.502)	0.402 (0.269–0.541)		
162	Fracture, other than femoral neck (short term, with or without treatment)	0.233 (0.159–0.324)	0.111 (0.074–0.156)	Japan > GBD	
163	Fracture, other than femoral neck (long term, without treatment)	0.079 (0.052–0.113)	0.042 (0.027–0.063)		
164	Fracture of patella, tibia or fibula or ankle (short term, with or without treatment)	0.213 (0.143–0.294)	0.050 (0.032–0.075)	Japan > GBD	Japan > GBD
165	Fracture of patella, tibia or fibula or ankle (long term, with or without treatment)	0.122 (0.083–0.170)	0.055 (0.036–0.081)	Japan > GBD	
166	Fracture of pelvis (short term)	0.431 (0.296–0.577)	0.279 (0.188–0.384)		
167	Fracture of pelvis (long term)	0.143 (0.098–0.204)	0.182 (0.123–0.253)		
168	Fracture of radius or ulna (short term, with or without treatment)	0.081 (0.054–0.118)	0.028 (0.016–0.046)	Japan > GBD	
169	Fracture of radius or ulna (long term, without treatment)	0.079 (0.052–0.111)	0.043 (0.028–0.064)		
170	Fracture of skull (short or long term, with or without treatment)	0.132 (0.087–0.190)	0.071 (0.048–0.100)		
171	Fracture of sternum and/or fracture of one or two ribs (short term, with or without treatment)	0.170 (0.116–0.238)	0.103 (0.068–0.145)		
172	Fracture of vertebral column (short or long term, with or without treatment)	0.106 (0.070–0.147)	0.111 (0.075–0.156)		
173	Fractures, treated (long term)	0.008 (0.003–0.017)	0.005 (0.002–0.014)		
174	Injured nerves (short term)	0.237 (0.157–0.329)	0.100 (0.067–0.140)	Japan > GBD	
175	Injured nerves (long term)	0.161 (0.111–0.223)	0.113 (0.076–0.157)		
176	Injury to eyes (short term)	0.076 (0.049–0.109)	0.054 (0.035–0.081)		
177	Concussion	0.170 (0.112–0.244)	0.110 (0.074–0.158)		
178	Severe traumatic brain injury, short term (with or without treatment)	0.114 (0.075–0.162)	0.214 (0.141–0.297)		
179	Traumatic brain injury, long-term consequences, minor (with or without treatment)	0.192 (0.132–0.266)	0.094 (0.063–0.133)	Japan > GBD	
180	Traumatic brain injury, long-term consequences, moderate (with or without treatment)	0.212 (0.144–0.299)	0.231 (0.156–0.324)		
181	Traumatic brain injury, long-term consequences, severe (with or without treatment)	0.455 (0.315–0.600)	0.637 (0.462–0.789)		
182	Open wound (short term, with or without treatment)	0.035 (0.021–0.055)	0.006 (0.002–0.012)	Japan > GBD	Japan > GBD
183	Poisoning (short term with or without treatment)	0.276 (0.193–0.377)	0.163 (0.109–0.227)		
184	Severe chest injury (long term, with or without treatment)	0.065 (0.042–0.095)	0.047 (0.030–0.070)		
185	Severe chest injury (short term, with or without treatment)	0.220 (0.152–0.306)	0.369 (0.248–0.501)		
186	Spinal cord lesion below neck level (treated)	0.388 (0.270–0.524)	0.296 (0.198–0.414)		
187	Spinal cord lesion below neck level (untreated)	0.564 (0.404–0.722)	0.623 (0.434–0.777)		
188	Spinal cord lesion at neck level (treated)	0.637 (0.468–0.792)	0.589 (0.415–0.748)		
189	Spinal cord lesion at neck level (untreated)	0.707 (0.527–0.842)	0.732 (0.544–0.871)		
Other					
190	Abdominopelvic problem, mild	0.029 (0.016–0.046)	0.011 (0.005–0.021)	Japan > GBD	
191	Abdominopelvic problem, moderate	0.392 (0.270–0.524)	0.114 (0.078–0.159)	Japan > GBD	Japan > GBD
192	Abdominopelvic problem, severe	0.339 (0.235–0.458)	0.324 (0.220–0.442)		
193	Anemia, mild	0.004 (0.001–0.009)	0.004 (0.001–0.008)		
194	Anemia, moderate	0.064 (0.040–0.092)	0.052 (0.034–0.076)		
195	Anemia, severe	0.040 (0.024–0.061)	0.149 (0.101–0.209)	Japan < GBD	Japan < GBD
196	Periodontitis	0.008 (0.003–0.015)	0.007 (0.003–0.014)		
197	Dental caries: symptomatic	0.035 (0.021–0.053)	0.010 (0.005–0.019)	Japan > GBD	Japan > GBD
198	Severe tooth loss	0.082 (0.053–0.115)	0.067 (0.045–0.095)		
199	Disfigurement: level 1	0.043 (0.026–0.063)	0.011 (0.005–0.021)	Japan > GBD	Japan > GBD
200	Disfigurement: level 2	0.123 (0.083–0.171)	0.067 (0.044–0.096)		
201	Disfigurement: level 3	0.512 (0.362–0.670)	0.405 (0.275–0.546)		
202	Generic uncomplicated disease: worry and daily medication	0.016 (0.008–0.028)	0.049 (0.031–0.072)	Japan < GBD	Japan < GBD
203	Generic uncomplicated disease: anxiety about diagnosis	0.008 (0.003–0.015)	0.012 (0.006–0.023)		
204	Severe wasting	0.086 (0.056–0.124)	0.128 (0.082–0.183)		
205	Speech problems	0.065 (0.041–0.094)	0.051 (0.032–0.078)		
206	Motor impairment, mild	0.009 (0.004–0.016)	0.010 (0.005–0.019)		
207	Motor impairment, moderate	0.045 (0.028–0.065)	0.061 (0.040–0.089)		
208	Motor impairment, severe	0.294 (0.202–0.396)	0.402 (0.268–0.545)		
209	Motor plus cognitive impairments, mild	0.023 (0.013–0.038)	0.031 (0.018–0.050)		
210	Motor plus cognitive impairments, moderate	0.106 (0.069–0.150)	0.203 (0.134–0.290)		
211	Motor plus cognitive impairments, severe	0.457 (0.318–0.606)	0.542 (0.374–0.702)		
212	Thrombocytopenic purpura	0.110 (0.073–0.154)	0.159 (0.106–0.226)		
213	Hypothyroidism	0.012 (0.005–0.023)	0.019 (0.010–0.032)		
214	Hyperthyroidism	0.103 (0.069–0.146)	0.145 (0.096–0.202)		
215	Vertigo and balance disorder (Menière, labyrinthitis)	0.102 (0.069–0.145)	0.113 (0.074–0.158)		
216	Allergic rhinitis (hay fever)	0.009 (0.004–0.016)	0.007 (0.003–0.017)		
217	Borderline personality disorder	0.132 (0.091–0.186)	0.190 (0.120–0.262)		
218	Carpal tunnel syndrome	0.020 (0.011–0.034)	0.035 (0.023–0.055)		
219	Constipation	0.048 (0.030–0.070)	0.061 (0.040–0.093)		
220	Hemorrhoids	0.090 (0.058–0.127)	0.109 (0.072–0.154)		
221	Heart burn and reflux “GERD”	0.046 (0.029–0.069)	0.027 (0.015–0.046)		
222	Insomnia	0.036 (0.022–0.055)	0.016 (0.009–0.031)	Japan > GBD	
223	Intensive care unit admission	0.675 (0.506–0.822)	0.739 (0.526–0.891)		
224	Invasive device/drain	0.512 (0.362–0.664)	0.143 (0.095–0.207)	Japan > GBD	Japan > GBD
225	Irritable bowel syndrome	0.039 (0.024–0.059)	0.064 (0.040–0.093)		
226	Sleep apnea	0.024 (0.014–0.040)	0.032 (0.020–0.051)		
227	Somatoform disorder	0.060 (0.038–0.086)	0.140 (0.095–0.199)	Japan < GBD	
228	Varicose veins	0.018 (0.010–0.031)	0.020 (0.010–0.037)		
229	Trigeminal neuralgia	0.128 (0.086–0.181)	0.068 (0.045–0.101)		
230	Vaginal discharge	0.096 (0.062–0.135)	NA		
231	Dermatitis	0.079 (0.050–0.115)	NA		

*GBD* Global Burden of Disease study, *DW* Disability weigh

**Fig. 3:**
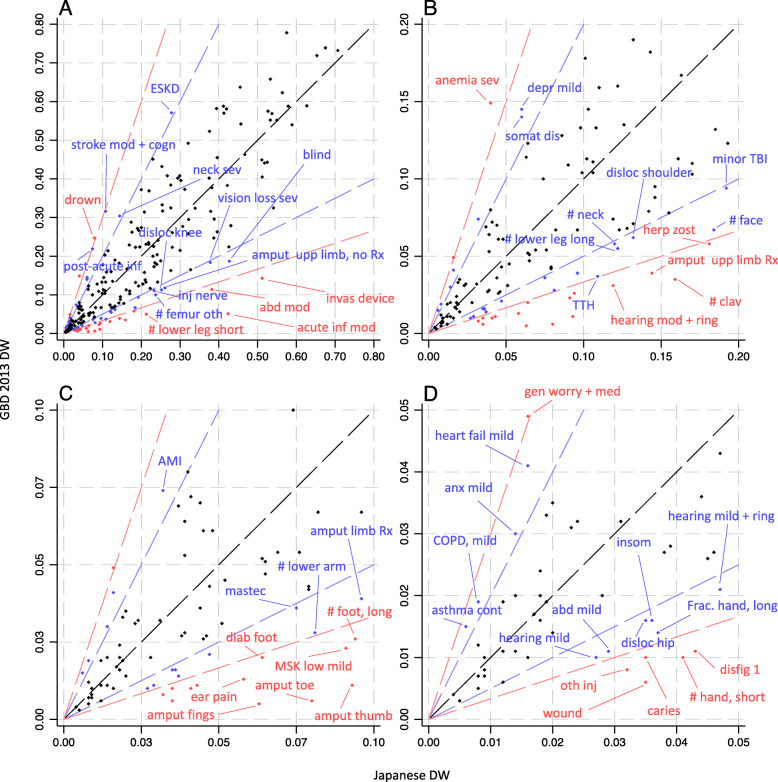
Comparison of Japanese disability weights and GBD 2013 disability weights: (**a**) all values; (**b**) zoomed in on values <0.2; (**c**) zoomed in on values <0.1; (**d**) zoomed in on values<0.05. The black line is a diagonal line, representing equivalence between Japanese and GBD 2013 disability weights. The blue line represents a factor-of-two difference, and the red line represents a factor-of-three difference. abd mild: abdominopelvic problem, mild; abd mod: abdominopelvic problem, moderate; AMI: acute myocardial infarction, days 3-28; amput fings: amputation of finger(s), excluding thumb; amput limb Rx: amputation of one lower limb (long term, with treatment); amput thumb: amputation of thumb (long term); amput toe: amputation of toe; amput upp limb Rx: amputation of one upper limb (with treatment); amput upp limb, no Rx: amputation of one upper limb (long term, without treatment); anemia sev: anemia, severe; anx mild: anxiety disorders, mild; asthma cont: asthma, controlled; COPD, mild: COPD and other chronic respiratory problems, mild; caries: dental caries: symptomatic; depr mild: major depressive disorder, mild episode; diab foot: diabetic foot; disfig 1: disfigurement: level 1; disloc hip: dislocation of hip (long term, with or without treatment); disloc knee: dislocation of knee (long term, with or without treatment); disloc shoulder: dislocation of shoulder (long term, with or without treatment); blind: distance vision blindness; vision loss sev: distance vision, severe impairment; drown: drowning and nonfatal submersion (short or long term, with or without treatment); ear pain: ear pain; ESKD: end-stage kidney disease, on dialysis; # clav: fracture of clavicle, scapula or humerus (short or long term, with or without treatment); # face: fracture of face bone (short or long term with or without treatment); # foot, long: fracture of foot bones (short term, with or without treatment); # hand, short: fracture of hand (short term, with or without treatment); # neck: fracture of neck of femur (long term, with treatment); # femur oth: fracture, other than femoral neck (short term, with or without treatment); # lower leg short: fracture of patella, tibia or fibula or ankle (short term, with or without treatment); # lower leg long: fracture of patella, tibia or fibula or ankle (long term, with or without treatment); # lower arm: fracture of radius or ulna (short term, with or without treatment); gen worry + med: generic uncomplicated disease: worry and daily medication.; TTH: tension-type headache; heart fail mild: heart failure, mild; hearing mild + ring: hearing loss, mild, with ringing; hearing mild: hearing loss, mild; hearing mod + ring: hearing loss, moderate, with ringing; herp zost: herpes zoster; acute inf mod: infectious disease, acute episode, moderate; post-acute inf : infectious disease, post-acute consequences (fatigue, emotional lability, insomnia); inj nerve: injured nerves (short term); insom: insomnia; invas device: invasive device/drain; mastec: mastectomy; MSK low mild: musculoskeletal problems, lower limbs, mild; neck sev: neck pain, chronic, severe; wound: open wound (short term, with or without treatment); oth inj: Other injuries of muscle and tendon (includes sprains, strains and dislocations other than shoulder, knee, hip); somat dis: somatoform disorder; stroke mod + cogn: stroke, long-term consequences, moderate plus cognition problems; minor TBI: Traumatic brain injury, long-term consequences, minor (with or without treatment)

Overall, a high correlation between Japanese DW and GBD 2013 DW was observed (0.88 for Spearman’s correlation coefficient), although there was considerable disagreement. Figure 3 shows a scatter plot of the Japanese and GBD 2013 DWs. The blue and red lines are straight lines that present the difference by a factor of two and three in the Japanese DW and GBD 2013 DW, respectively. Out of 226 health states, 55 (24.3%) showed more than a twofold difference, of which 41 (74.6%) had a higher value in Japanese DW. More than a factor-of-three difference was found for 23 health states (13.0%), of which 20 (87.0%) were health states with higher DW in Japan and they were mostly injuries including amputations and fractures. The largest proportional difference was a 13 times higher Japanese DW for amputation of toe (0.080 [95% UI 0.052–0.114]) compared to the GBD DW of 0.006 [0.002–0.012]), followed by a 12 times higher Japanese DW for amputation of finger(s), excluding thumb (0.063 [0.041–0.092] vs 0.005 [0.002–0.010]), an 8.5 times higher Japanese DW for amputation of thumb (long term) (0.093 [0.061–0.132] vs 0.011 [0.005–0.021]), and 8.3 times higher Japanese DW for moderate acute infectious disease (0.424 [0.289–0.577] vs 0.051 [0.032–0.074]). On the other hand, for the following three health states, the GBD 2013 DWs were higher than the Japanese DWs by a factor of three or more: drowning (0.079 [0.052–0.114] vs 0.247 [0.164–0.341]); severe anemia (0.040 [0.024–0.061] vs 0.149 [0.101–0.209]); generic uncomplicated disease: worry and daily medication (0.016 [0.008–0.028] vs 0.049 [0.031–0.072]).

The distribution of the difference between the Japanese DW and GBD 2013 DW is presented in Additional file 1: figure 1, and the health category-specific differences are shown in Additional file 1: figures 2–12. Remarkable differences were found in several health categories. Japanese DWs for injuries and hearing and vision loss were generally larger than the GBD 2013 DW, whereas mental, behavioral, and substance use disorder were generally larger in the GBD 2013 DW than in the Japanese DW.

We found an inconsistency of DWs in four out of 28 diseases and injuries with a gradient in severity between health states. This concerned infectious disease episodes, neck pain, abdominopelvic problem, and anemia. Moderate infectious disease episode had a higher DW (0.424 [95% UI 0.289–0.577]) than severe infectious disease episode (0.242 [0.163–0.340]); severe neck pain had a higher DW (0.169 [0.115–0.236]) than most severe neck pain (0.144 [0.099–0.200]); moderate abdominopelvic problem had a higher DW (0.382 [0.270–0.524]) than severe moderate abdominopelvic problem (0.339 [0.235–0.458]); and moderate anemia had a higher DW (0.064 [0.040–0.092]) than severe anemia (0.040 [0.024–0.061]). All comparisons of conditions with several severity levels are presented in Additional file 1: figure 13.

The results of the regression analysis by key symptoms mentioned in the lay descriptions are shown in Additional file 1: table 2. Mental symptoms, substance use, and the residual category of other physical symptoms were statistically significantly associated with a lower Japanese DW than the GBD 2013 DW. The symptoms of pain and sensory symptoms were statistically significantly associated with a higher Japanese DW than the GBD 2013 DW. These findings remained robust in sensitivity analyses with the exclusion of non-significant symptoms.

## Discussion

Disease burden research is primarily used as a decision-making tool to prioritize resource allocation at the population level, and it has been recommended to incorporate the health perceptions of the public in order to inform decision-making in democratic societies [3, 16–18]. In Japan, however, there has not been a comprehensive assessment of health states based on valuations by the general population. Burden of disease assessment in Japan has relied on the GBD studies in other countries. We found considerable disagreement between Japanese DWs and GBD DWs. Health states for injuries, and hearing and vision loss were valued as more severe and mental, and substance use disorders were less severe in Japan. Health states with pain and sensory symptoms in the lay descriptions were significantly valued higher in our study while mental symptoms, substance use, and a residual category of other physical symptoms had higher DWs in GBD.

### Differences of estimated Japanese DW from the GBD 2013 DW

Like the GBD 2010 and the European DW study, the present study aimed to quantify the severity of health loss, rather than general welfare loss. Many previous studies have shown that there are clear contextual differences (such as socioeconomic status, ethnicity, and living environment) in how people perceive health problems and how such problems affect their lives [6, 19–27]. For instance, Komiyama et al. found that Japanese people were more sensitive to pain-related suffering when some pain detection thresholds were compared with Belgians and Caucasians [22, 23].

Tsuchiya et al. pointed out that the EQ-5D instrument, which was developed based on the health perspectives in European settings as a measure of health-related quality of life, does not necessarily adequately assess that of the Japanese [28]. Gerlinger et al. reported that the value sets of the EQ-5D-5L utility index between Denmark, France, Germany, Japan, Netherlands, Spain, Thailand, UK, US, and Zimbabwe varied substantially. They argued that when analyzing multinational clinical trials, country-specific value sets should be used to assess treatment effects on patient health perceptions [29, 30].

Ustün et al. also showed a significant difference in the disability rank of health conditions in 14 countries (Canada, China, Egypt, Greece, India, Japan, Luxembourg, Netherlands, Nigeria, Romania, Spain, Tunisia, Turkey, and the UK) [6]. The study included a total of 241 health professionals, policy makers, and patients, who subjectively ranked 17 health conditions from most disabling to least disabling. For Japan, the ranking of amputation and blindness was relatively high compared to other countries, while major depression and drug dependence were relatively lowly ranked, analogous to our findings. In the present study, the Japanese DW was higher than the GBD 2013 DW in all states related to amputation, especially the amputation of toe, which differed by a factor of 13. Similar differences in the Ustün study were found in China, but not in the UK, Canada, and other European countries. Also, in a 2016 DW study in South Korea, the DW of injuries, and hearing and vision loss were estimated to be considerably larger than those of GBD 2010 DW. However, this study modified the study protocol compromising the ability to make direct comparisons [7].

In the DW studies incorporated into GBD, the pair-wise comparisons of different health states produced similar results in different cultural, educational, environmental, and demographic contexts [3, 5]. However, it should be noted that the majority of responses came from high-income countries and around a quarter from four low- and middle-income countries, raising concerns about the universality of the DW estimates. This study, with a sample size two-thirds of the combined set of responses from the GBD 2010 and European DW study, shows enough differences in the DW values to challenge the universality of DWs as applied in GBD. However, only few studies are available that would allow contextual examination of differences in DWs across a wide range of health states but these studies have been conducted in a more distant past and used very different methods [7, 18, 19, 31]. There clearly is a need for further comparable studies to address the contextual differences. The differences between the GBD and Japanese DWs also raise the question whether one set of disability weights should be used universally, as is now the practice for the GBD study, or whether country-specific DWs should be used in future iterations of the GBD. Using a universal set of DWs has the great advantage of allowing country comparisons of burden of diseases in a standardized manner across countries, and is very useful for identifying drivers of successes and failures in health improvement of specific countries. On the other hand, countries may choose their own disability weights to better reflect preferences of their population. We would advise the GBD incorporates our findings in a new joint analysis with all previous studies and thus reduce the gap between the Japanese and previous GBD DWs for future iterations.

We also recommend that future DW studies cover the populations that are not represented in the GBD 2013 DWs. Note that the GBD 2013 DWs relied on sampling from the original GBD 2010 DW study and the subsequent European study. The GBD 2010 DW study was based on surveys in four low- and middle-income countries and five high-income countries supplemented by a web-based survey with respondents from many countries but the majority coming from North America, Australia, Western Europe and, to a lesser extent from China, India, Brazil, and South Africa. Data from most other countries were rather limited [3]. The target audience of the surveys was also limited to those aged 18 years or older. Meanwhile, the population of the European DW study consisted of those aged 18 to 65 years from four European countries, namely, Hungary, Italy, the Netherlands, and Sweden [5]. There is a large data gap in countries that were not covered by these studies, and we expect that future DW studies address this gap, which will help to contribute to the methodological and empirical basis for the modeling framework in future iterations of the GBD.

### Strengths and limitations

The strength of this study was the large number of respondents. The size of the sample allowed for detailed estimation of DWs within the Japanese context. Meanwhile, the use of a web-based survey for data collection constituted limitations of our study. Internet users tend to be more highly educated and younger than the general Japanese population, limiting interpretation of our findings as being fully reflective of the opinions of the Japanese population.

The responses of PC had high consistency for each pair of health states when viewed in the heatmap. The 21% disagreement in the test-retest assessment was largely similar to the findings of previous studies [5]. However, we found an inconsistency of DWs in four out of 28 diseases and injuries that had several health states of increasing severity. This inconsistency may be explained by the expression of Japanese translation that the lay description of these health states did not capture the intended difference of level of severity. In this regard, many approaches have been discussed in literature to improve the validity and reliability of translated questionnaires [32–35]. Literature reviews proposed that in addition to the linguistic equivalence (which we ensured using the forward/backward-translation technique in the present study), the cultural adaptation of the original questionnaire needs to be explored [32–34]. They suggested conducting pilot testing prior to survey launch to assess the cultural equivalence, such as if the meanings of questionnaire items in the written language is viewed and interpreted in the intended way, by means of interviews with representatives of prospective respondents, followed by evaluation of psychometric properties using different tests.

The responses of PHE questions showed no variation between health states that did vary widely in the pair wise comparisons. The European DW study reported a similar lack of discernment in the PHE responses and speculated that the cognitive demands of the PHE questions was better suited to the GBD internet panel consisting largely of tertiary educated health professionals, rather than general population samples [3, 5]. As the proportion of respondents with higher education in our sample was substantially higher than the general population (46%), the more important reason for the greater success of the PHE questions in the GBD survey may be that respondents were a self-selected group who were evidently interested in the content of the survey and voluntarily participated. This may have improved the signal-to-noise ratio in their response. Our survey, on the other hand, was conducted by participants randomly selected from an existing panel and given incentives (points). This may have affected the attention paid to the intent of this study or the amount of time to consider more complex questions. We concluded that PHE questions might not be suitable for a web-based survey among the general population because of the high cognitive demand to make a meaningful distinction between the two hypothetical health programs.

## Conclusions

This study has provided an empirical basis for DWs that are specific to Japan. Despite high correlation, considerable disagreement between Japanese DWs and GBD 2013 DWs were observed. Our findings suggest sizeable cultural differences in perceptions of the severity of key domains of ill health among the Japanese with greater severity assigned to pain and sensory loss but lower severity to mental and substance use disorders. The ramifications are that for resource allocation decision-making in Japan, this set of DWs may be more appropriate than the GBD DWs. However, for international comparisons of disease burden, it remains desirable to continue using a common set of DWs. For future rounds of the GBD study, combined analysis of all previous GBD pair wise comparison results with this new information from Japan is recommended.

## Additional file

**Additional file 1: Appendix Figure 1.** Distribution of the absolute difference (top panel) and % difference (bottom panel) between the Japanese DW and GBD 2013 DW (*n*=226). **Appendix Figure 2.** Distribution of the absolute difference (top panel) and % difference (bottom panel) between the Japanese DW and GBD 2013 DW for infectious disease (*n*=15). **Appendix Figure 3.** Distribution of the absolute difference (top panel) and % difference (bottom panel) between the Japanese DW and GBD 2013 DW for cancer (*n*=6). **Appendix Figure 4.** Distribution of the absolute difference (top panel) and % difference (bottom panel) between the Japanese DW and GBD 2013 DW for cardiovascular and circulatory disease (*n*=15). **Appendix Figure 5.** Distribution of the absolute difference (top panel) and % difference (bottom panel) between the Japanese DW and GBD 2013 DW for diabetes and digestive and genitourinary disease (*n*=14). **Appendix Figure 6.** Distribution of the absolute difference (top panel) and % difference (bottom panel) between the Japanese DW and GBD 2013 DW for chronic respiratory disease (*n*=6). **Appendix Figure 7.** Distribution of the absolute difference (top panel) and % difference (bottom panel) between the Japanese DW and GBD 2013 DW for neurological disorders (*n*=14). **Appendix Figure 8.** Distribution of the absolute difference (top panel) and % difference (bottom panel) between the Japanese DW and GBD 2013 DW for mental, behavioural, and substance use disorders (*n*=25). **Appendix Figure 9.** Distribution of the absolute difference (top panel) and % difference (bottom panel) between the Japanese DW and GBD 2013 DW for hearing and vision loss (*n*=15). **Appendix Figure 10.** Distribution of the absolute difference (top panel) and % difference (bottom panel) between the Japanese DW and GBD 2013 DW for musculoskeletal disorders (*n*=18). **Appendix Figure 11.** Distribution of the absolute difference (top panel) and % difference (bottom panel) between the Japanese DW and GBD 2013 DW for injuries (*n*=58). **Appendix Figure 12.** Distribution of the absolute difference (top panel) and % difference (bottom panel) between the Japanese DW and GBD 2013 DW for others (*n*=40). **Appendix Figure 13.** Comparison of level of severity and Japanese DW by health state. **Appendix Table 1.** Lay descriptions for 231 health states and symptom categories. **Appendix Table 2.** Regression analysis results for differences between the Japanese DW and GBD 2013 DW for 226 comparable health states.

## Data Availability

The datasets used and/or analyzed during the current study are available from the corresponding author on reasonable request.

## Abbreviations

GBD: Global burden of disease
DALYs: Disability-adjusted life years
YLLs: Years of life lost
YLDs: Years lived with disability
DW: Disability weight
PC: Paired comparison
PHE: Population health equivalence
UI: Uncertainty intervals
ADL: Activities of daily living
