# Supplementary material for: How do Japanese rate the severity of different diseases and injuries?—an assessment of disability weights for 231 health states by 37,318 Japanese respondents
Source: Popul Health Metr. 2021 Apr 23;19:21. doi: 10.1186/s12963-021-00253-4 (PMC8063365; doi:10.1186/s12963-021-00253-4)
Supplement: Supplementary file 1 — Additional file 1: Appendix Figure 1. Distribution of the absolute difference (top panel) and % difference (bottom panel) between the Japanese DW and GBD 2013 DW (n=226). Appendix Figure 2. Distribution of the absolute difference (top panel) and % difference (bottom panel) between the Japanese DW and GBD 2013 DW for infectious disease (n=15). Appendix Figure 3. Distribution of the absolute difference (top panel) and % difference (bottom panel) between the Japanese DW and GBD 2013 DW for cancer (n=6). Appendix Figure 4. Distribution of the absolute difference (top panel) and % difference (bottom panel) between the Japanese DW and GBD 2013 DW for cardiovascular and circulatory disease (n=15). Appendix Figure 5. Distribution of the absolute difference (top panel) and % difference (bottom panel) between the Japanese DW and GBD 2013 DW for diabetes and digestive and genitourinary disease (n=14). Appendix Figure 6. Distribution of the absolute difference (top panel) and % difference (bottom panel) between the Japanese DW and GBD 2013 DW for chronic respiratory disease (n=6). Appendix Figure 7. Distribution of the absolute difference (top panel) and % difference (bottom panel) between the Japanese DW and GBD 2013 DW for neurological disorders (n=14). Appendix Figure 8. Distribution of the absolute difference (top panel) and % difference (bottom panel) between the Japanese DW and GBD 2013 DW for mental, behavioural, and substance use disorders (n=25). Appendix Figure 9. Distribution of the absolute difference (top panel) and % difference (bottom panel) between the Japanese DW and GBD 2013 DW for hearing and vision loss (n=15). Appendix Figure 10. Distribution of the absolute difference (top panel) and % difference (bottom panel) between the Japanese DW and GBD 2013 DW for musculoskeletal disorders (n=18). Appendix Figure 11. Distribution of the absolute difference (top panel) and % difference (bottom panel) between the Japanese DW and GBD 2013 DW for injuries (n=58). Append [file 12963_2021_253_MOESM1_ESM.docx]

**Appendix figure 1: Distribution of the absolute difference (top panel) and % difference (bottom panel) between the Japanese DW and GBD 2013 DW (n=226)**

GBD: Global Burden of Disease study; DW: disability weight; Blue: Japanese DW > GBD 2013 DW; Red: GBD 2013 DW > Japanese DW; Black: Japanese DW = GBD 2013 DW (i.e. the value is the same when rounding the third decimal place).

**Appendix figure 2. Distribution of the absolute difference (top panel) and % difference (bottom panel) between the Japanese DW and GBD 2013 DW for infectious disease (n=15)**

GBD: Global Burden of Disease study; DW: disability weight; Blue: Japanese DW > GBD 2013 DW; Red: GBD 2013 DW > Japanese DW.

**Appendix figure 3. Distribution of the absolute difference (top panel) and % difference (bottom panel) between the Japanese DW and GBD 2013 DW for cancer (n=6)**

GBD: Global Burden of Disease study; DW: disability weight; Blue: Japanese DW > GBD 2013 DW; Red: GBD 2013 DW > Japanese DW.

**Appendix figure 4. Distribution of the absolute difference (top panel) and % difference (bottom panel) between the Japanese DW and GBD 2013 DW for cardiovascular and circulatory disease (n=15)**

GBD: Global Burden of Disease study; DW: disability weight; Blue: Japanese DW > GBD 2013 DW; Red: GBD 2013 DW > Japanese DW; Black: Japanese DW = GBD 2013 DW (i.e. the value is the same when rounding the third decimal place).

**Appendix figure 5. Distribution of the absolute difference (top panel) and % difference (bottom panel) between the Japanese DW and GBD 2013 DW for diabetes and digestive and genitourinary disease (n=14)**

GBD: Global Burden of Disease study; DW: disability weight; Blue: Japanese DW > GBD 2013 DW; Red: GBD 2013 DW > Japanese DW; Black: Japanese DW = GBD 2013 DW (i.e. the value is the same when rounding the third decimal place).

**Appendix figure 6. Distribution of the absolute difference (top panel) and % difference (bottom panel) between the Japanese DW and GBD 2013 DW for chronic respiratory disease (n=6)**

GBD: Global Burden of Disease study; DW: disability weight; Blue: Japanese DW > GBD 2013 DW; Red: GBD 2013 DW > Japanese DW.

**Appendix figure 7. Distribution of the absolute difference (top panel) and % difference (bottom panel) between the Japanese DW and GBD 2013 DW for neurological disorders (n=14)**

GBD: Global Burden of Disease study; DW: disability weight; Blue: Japanese DW > GBD 2013 DW; Red: GBD 2013 DW > Japanese DW.

**Appendix figure 8. Distribution of the absolute difference (top panel) and % difference (bottom panel) between the Japanese DW and GBD 2013 DW for mental, behavioural, and substance use disorders (n=25)**

GBD: Global Burden of Disease study; DW: disability weight; Blue: Japanese DW > GBD 2013 DW; Red: GBD 2013 DW > Japanese DW.

**Appendix figure 9. Distribution of the absolute difference (top panel) and % difference (bottom panel) between the Japanese DW and GBD 2013 DW for hearing and vision loss (n=15)**

GBD: Global Burden of Disease study; DW: disability weight; Blue: Japanese DW > GBD 2013 DW; Red: GBD 2013 DW > Japanese DW.

**Appendix figure 10. Distribution of the absolute difference (top panel) and % difference (bottom panel) between the Japanese DW and GBD 2013 DW for musculoskeletal disorders (n=18)**

GBD: Global Burden of Disease study; DW: disability weight; Blue: Japanese DW > GBD 2013 DW; Red: GBD 2013 DW > Japanese DW.

**Appendix figure 11. Distribution of the absolute difference (top panel) and % difference (bottom panel) between the Japanese DW and GBD 2013 DW for injuries (n=58)**

GBD: Global Burden of Disease study; DW: disability weight; Blue: Japanese DW > GBD 2013 DW; Red: GBD 2013 DW > Japanese DW.

**Appendix figure 12. Distribution of the absolute difference (top panel) and % difference (bottom panel) between the Japanese DW and GBD 2013 DW for others (n=40)**

GBD: Global Burden of Disease study; DW: disability weight; Blue: Japanese DW > GBD 2013 DW; Red: GBD 2013 DW > Japanese DW; Black: Japanese DW = GBD 2013 DW (i.e. the value is the same when rounding the third decimal place).

**Appendix figure 13. Comparison of level of severity and Japanese DW by health state**

DW: disability weight; MSK: Musculoskeletal; TBI: Traumatic brain injury.

**Appendix table 1: Lay descriptions for 231 health states and symptom categories**

| Id | Category | Health state | Lay description | Chronic or acute | | Symptoms | | | | | | | | | | |
| --- | --- | --- | --- | --- | --- | --- | --- | --- | --- | --- | --- | --- | --- | --- | --- | --- |
|  |  |  |  | Chronic | Acute | Mobility | Pain | Mental symptom | Fatigue | Disfigurement | Sensory symptom | Infection/  diarrhea | Substance  use | ADL | Cognition | Others |
| Infectious disease | | |  |  |  |  |  |  |  |  |  |  |  |  |  |  |
| 1 | GBD 2010 original | Infectious disease, acute episode, mild | has a low fever and mild discomfort, but no difficulty with daily activities. |  | * |  |  |  |  |  |  | ○ |  |  |  |  |
| 2 | GBD 2010 original | Infectious disease, acute episode, moderate | has a fever and aches, and feels weak, which causes some difficulty with daily activities. |  | * |  | ○ |  | ○ |  |  | ○ |  | ○ |  |  |
| 3 | GBD 2010 original | Infectious disease, acute episode, severe | has a high fever and pain, and feels very weak, which causes great difficulty with daily activities. |  | * |  | ○ |  | ○ |  |  | ○ |  | ○ |  |  |
| 4 | GBD 2010 original | Infectious disease, post-acute consequences (fatigue, emotional lability, insomnia) | is always tired and easily upset. The person feels pain all over the body and is depressed. |  | * |  | ○ | ○ | ○ |  |  |  |  |  |  |  |
| 5 | GBD 2010 original | Diarrhea, mild | has diarrhea three or more times a day with occasional discomfort in the belly. |  | * |  |  |  |  |  |  | ○ |  |  |  |  |
| 6 | GBD 2010 original | Diarrhea, moderate | has diarrhea three or more times a day, with painful cramps in the belly and feeling thirsty |  | * |  | ○ |  |  |  |  | ○ |  |  |  |  |
| 7 | GBD 2010 original | Diarrhea, severe | has diarrhea three or more times a day with severe belly cramps. The person is very thirsty and feels nauseous and tired. |  | * |  | ○ |  | ○ |  |  | ○ |  |  |  |  |
| 8 | GBD 2010 original | Epididymo-orchitis | has swelling and tenderness in the testicles and pain during urination. |  | * |  | ○ |  |  |  |  | ○ |  |  |  |  |
| 9 | GBD 2010 original | Herpes zoster | has a blistering skin rash that causes pain, with some burning and itching. |  | * |  | ○ |  |  |  |  |  |  |  |  |  |
| 10 | GBD 2010 original | HIV cases, symptomatic, pre-AIDS | has weight loss, fatigue, and frequent infections. | * |  |  | ○ |  | ○ |  |  | ○ |  |  |  |  |
| 11 | GBD 2010 original | HIV/AIDS cases, receiving ARV treatment | has occasional fevers and infections. The person takes daily medication that sometimes causes diarrhea. | * |  |  | ○ |  |  |  |  | ○ |  |  |  |  |
| 12 | GBD 2010 original | AIDS cases, not receiving ARV treatment | has severe weight loss, weakness, fatigue, cough and fever, and frequent infections, skin rashes and diarrhea. | * |  |  |  |  | ○ | ○ |  | ○ |  |  |  | ○ |
| 13 | GBD 2010 original | Ear pain | has an ear-ache that causes some difficulty with daily activities. |  | * |  | ○ |  |  |  |  |  |  | ○ |  |  |
| 14 | GBD 2010 original | Tuberculosis, HIV infected | has a persistent cough and fever, shortness of breath, night sweats, weakness and fatigue and severe weight loss. | * |  |  |  |  | ○ |  |  | ○ |  |  |  |  |
| 15 | GBD 2010 original | Tuberculosis, not HIV infected | has a persistent cough and fever, is short of breath, feels weak, and has lost a lot of weight. | * |  |  |  |  |  |  |  |  |  |  |  |  |
| Cancer | | |  |  |  |  |  |  |  |  |  |  |  |  |  |  |
| 16 | GBD 2010 original | Cancer, diagnosis and primary therapy | has pain, nausea, fatigue, weight loss and high anxiety. | * | * |  | ○ | ○ | ○ |  |  |  |  |  |  |  |
| 17 | GBD 2010 original | Cancer, metastatic | has severe pain, extreme fatigue, weight loss and high anxiety. | * | * |  | ○ | ○ | ○ |  |  |  |  |  |  |  |
| 18 | Japanese new | Cancer, after treatment | decreased physical strength due to treatment, feels tired and sometimes has abdominal pain or swelling of upper/lower limb. The person has regular examination and feels anxious until the result every time. | * |  |  | ○ | ○ | ○ |  |  |  |  |  |  |  |
| 19 | GBD 2010 original | Mastectomy | had one of her breasts removed and sometimes has pain or swelling in the arms. | * |  |  | ○ |  |  | ○ |  |  |  |  |  |  |
| 20 | GBD 2010 original | Stoma | has a pouch attached to an opening in the belly to collect and empty stools. | * |  |  |  |  |  | ○ |  |  |  |  |  |  |
| 21 | GBD 2010 original | Terminal phase, with medication (for cancers, end-stage kidney/liver disease) | has lost a lot of weight and regularly uses strong medication to avoid constant pain. The person has no appetite, feels nauseous, and needs to spend most of the day in bed. | * |  |  | ○ |  | ○ |  |  |  |  | ○ |  |  |
| 22 | GBD 2010 original | Terminal phase, without medication (for cancers, end-stage kidney/liver disease) | has lost a lot of weight and has constant pain. The person has no appetite, feels nauseous, and needs to spend most of the day in bed. | * |  |  | ○ |  | ○ |  |  |  |  | ○ |  |  |
| Cardiovascular and circulatory disease | | |  |  |  |  |  |  |  |  |  |  |  |  |  |  |
| 23 | GBD 2010 original | Acute myocardial infarction: days 1-2 | has severe chest pain that becomes worse with any physical activity,. The person feels nauseous, short of breath, and very anxious. |  | * |  | ○ |  |  |  |  |  |  |  |  | ○ |
| 24 | GBD 2010 original | Acute myocardial infarction, days 3-28 | gets short of breath after heavy physical activity, and tires easily, but has no problems when at rest. The person has to take medication every day and has some anxiety. | * | * |  |  | ○ | ○ |  |  |  |  |  |  | ○ |
| 25 | GBD 2010 original | Angina pectoris, mild | has chest pain that occurs with strenuous physical activity, such as running of lifting heavy objects. After a brief rest, the pain goes away. | * | * |  | ○ |  |  |  |  |  |  |  |  |  |
| 26 | GBD 2010 original | Angina pectoris, moderate | has chest pain that occurs with moderate physical activity, such as walking uphill or more than half a kilometer (around a quarter-mile) on level ground. After a brief rest, the pain goes away. | * | * |  | ○ |  |  |  |  |  |  |  |  |  |
| 27 | GBD 2010 original | Angina pectoris, severe | has chest pain that occurs with minimal physical activity, such as walking only a short distance. After a brief rest, the pain goes away. The person avoids most physical activities because of the pain. | * | * |  | ○ |  |  |  |  |  |  | ○ |  |  |
| 28 | GBD 2010 original | Cardiac conduction disorders and cardiac dysrhythmias | has periods of rapid and irregular heartbeats and occasional fainting. | * | * |  |  |  |  |  |  |  |  |  |  | ○ |
| 29 | GBD 2010 original | Claudication | has cramping pains in the legs after walking a medium distance. The pain goes away after a short rest. | * | * |  | ○ |  |  |  |  |  |  |  |  |  |
| 30 | GBD 2010 original | Heart failure, mild | is short of breath and easily tires with moderate physical activity, such as walking uphill or more than a quarter-mile on level ground. The person feels comfortable at rest or during activities requiring less effort. | * |  |  |  |  | ○ |  |  |  |  | ○ |  | ○ |
| 31 | GBD 2010 original | Heart failure, moderate | is short of breath and easily tires with minimal physical activity, such as walking only a short distance. The person feels comfortable at rest but avoids moderate activity. | * |  |  |  |  | ○ |  |  |  |  | ○ |  | ○ |
| 32 | GBD 2010 original | Heart failure, severe | is short of breath and feels tired when at rest. The person avoids any physical activity, for fear of worsening the breathing problems. | * |  |  |  |  | ○ |  |  |  |  | ○ |  | ○ |
| 33 | GBD 2010 original | Stroke: long-term consequences, mild | has some difficulty in moving around and some weakness in one hand, but is able to walk without help. | * |  | ○ |  |  |  |  |  |  |  |  |  |  |
| 34 | GBD 2010 original | Stroke: long-term consequences, moderate | has some difficulty in moving around, and in using the hands for lifting and holding things, dressing and grooming. | * |  | ○ |  |  |  |  |  |  |  |  |  |  |
| 35 | GBD 2010 original | Stroke: long-term consequences, moderate plus cognition problems | has some difficulty in moving around, in using the hands for lifting and holding things, dressing and grooming, and in speaking. The person is often forgetful and confused. | * |  | ○ |  |  |  |  |  |  |  |  | ○ |  |
| 36 | GBD 2010 original | Stroke: long-term consequences, severe | is confined to bed or a wheelchair, has difficulty speaking and depends on others for feeding, toileting and dressing. | * |  | ○ |  |  |  |  |  |  |  | ○ |  |  |
| 37 | GBD 2010 original | Stroke: long-term consequences, severe plus cognition problems | is confined to bed or a wheelchair, depends on others for feeding, toileting and dressing, and has difficulty speaking, thinking clearly and remembering things. | * |  | ○ |  |  |  |  |  |  |  | ○ | ○ |  |
| Diabetes and digestive and genitourinary disease | | |  |  |  |  |  |  |  |  |  |  |  |  |  |  |
| 38 | GBD 2010 original | Diabetic foot | has a sore on the foot that is swollen and causes some difficulty in walking. | * | * | ○ | ○ |  |  |  |  |  |  |  |  |  |
| 39 | GBD 2010 original | Diabetic neuropathy | has pain, tingling and numbness in the arms, legs, hands and feet. The person sometimes gets cramps and muscle weakness. | * | * | ○ | ○ |  |  |  |  |  |  |  |  |  |
| 40 | GBD 2010 original | Chronic kidney disease (stage IV) | tires easily, has nausea, reduced appetite and difficulty sleeping. | * | * |  |  |  | ○ |  |  |  |  |  |  | ○ |
| 41 | GBD 2010 original | End-stage renal disease, on dialysis | is tired and has itching, cramps, headache, joint pains and shortness of breath. The person needs intensive medical care every other day lasting about half a day. | * |  |  | ○ |  | ○ |  |  |  |  | ○ |  | ○ |
| 42 | GBD 2010 original | End-stage renal disease, with kidney transplant | sometimes feels tired and down, and has some difficulty with daily activities. | * | * |  |  |  | ○ |  |  |  |  | ○ |  |  |
| 43 | GBD 2010 original | Decompensated cirrhosis of the liver | has a swollen belly and swollen legs. The person feels weakness, fatigue and loss of appetite. | * |  |  |  |  | ○ |  |  |  |  |  |  | ○ |
| 44 | GBD 2010 original | Gastric bleeding | vomits blood and feels nauseous. |  | * |  |  |  |  |  |  |  |  |  |  | ○ |
| 45 | GBD 2010 original | Crohn's disease or ulcerative colitis | has cramping abdominal pain, has diarrhea several times a day, and feels very tired for two months every year. When the person does not have symptoms, there is anxiety about them returning. | * |  |  | ○ |  |  |  |  | ○ |  |  |  |  |
| 46 | GBD 2010 original | Benign prostatic hypertrophy: symptomatic | feels the urge to urinate frequently, but when passing urine it comes out slowly and sometimes is painful. | * | * |  | ○ |  |  |  |  |  |  |  |  | ○ |
| 47 | GBD 2010 original | Urinary incontinence | cannot control urinating. | * | * |  |  |  |  |  |  |  |  |  |  | ○ |
| 48 | European original | Stress incontinence | loses small amounts of urine without meaning to when coughing, sneezing, laughing or during physical exercise. | * | * |  |  |  |  |  |  |  |  |  |  | ○ |
| 49 | GBD 2010 original | Impotence | has difficulty in obtaining or maintaining an erection. | * | * |  |  |  |  |  |  |  |  |  |  | ○ |
| 50 | GBD 2010 original | Infertility, primary | wants to have a child and has a fertile partner, but the couple cannot conceive. | * |  |  |  |  |  |  |  |  |  |  |  | ○ |
| 51 | GBD 2010 original | Infertility, secondary | has at least one child, and wants to have more children. The person has a fertile partner, but the couple cannot conceive. | * |  |  |  |  |  |  |  |  |  |  |  | ○ |
| Chronic respiratory disease | | |  |  |  |  |  |  |  |  |  |  |  |  |  |  |
| 52 | GBD 2010 original | Asthma, controlled | has wheezing and cough once a month, which does not cause difficulty with daily activities. | * |  |  |  |  |  |  |  |  |  |  |  | ○ |
| 53 | GBD 2010 original | Asthma, partially controlled | has wheezing and cough once a week, which causes some difficulty with daily activities. | * |  |  |  |  |  |  |  |  |  | ○ |  | ○ |
| 54 | GBD 2010 original | Asthma: uncontrolled | has wheezing, cough and shortness of breath more than twice a week, which causes difficulty with daily activities and sometimes wakes the person at night. | * |  |  |  |  |  |  |  |  |  | ○ |  | ○ |
| 55 | GBD 2010 original | COPD and other chronic respiratory problems, mild | has cough and shortness of breath after heavy physical activity, but is able to walk long distances and climb stairs. | * | * |  |  |  |  |  |  |  |  |  |  | ○ |
| 56 | GBD 2010 original | COPD and other chronic respiratory problems, moderate | has cough, wheezing and shortness of breath, even after light physical activity. The person feels tired and can walk only short distances or climb only a few stairs. | * | * |  |  |  | ○ |  |  |  |  | ○ |  | ○ |
| 57 | GBD 2010 original | COPD and other chronic respiratory problems, severe | has cough, wheezing and shortness of breath all the time. The person has great difficulty walking even short distances or climbing any stairs, feels tired when at rest, and is anxious. | * | * |  |  | ○ | ○ |  |  |  |  | ○ |  | ○ |
| Neurological disorders | | |  |  |  |  |  |  |  |  |  |  |  |  |  |  |
| 58 | GBD 2010 original | Dementia, mild | has some trouble remembering recent events, and finds it hard to concentrate and make decisions and plans. | * |  |  |  |  |  |  |  |  |  | ○ | ○ |  |
| 59 | GBD 2010 original | Dementia, moderate | has memory problems and confusion, feels disoriented, at times hears voices that are not real, and needs help with some daily activities. | * |  |  |  | ○ |  |  |  |  |  | ○ | ○ |  |
| 60 | GBD 2010 original | Dementia, severe | has complete memory loss; no longer recognizes close family members; and requires help with all daily activities. | * |  |  |  |  |  |  |  |  |  | ○ | ○ |  |
| 61 | GBD 2010 original | Headache: migraine | has severe, throbbing head pain and nausea that cause great difficulty in daily activities and sometimes confine the person to bed. Moving around, light, and noise make it worse. | * | * |  | ○ |  |  |  |  |  |  | ○ |  |  |
| 62 | GBD 2010 original | Headache: tension-type | has a moderate headache that also affects the neck, which causes difficulty in daily activities. | * | * |  | ○ |  |  |  |  |  |  | ○ |  |  |
| 63 | GBD 2010 original | Headache: medication overuse | has daily headaches, felt as dull pain and often lasting all day, with poor sleep, nausea and fatigue. The person takes medicine for the headaches which provides little relief but is needed to avoid having worse symptoms. | * | * |  | ○ |  | ○ |  |  |  |  |  |  | ○ |
| 64 | GBD 2010 original | Multiple sclerosis, mild | has mild loss of feeling in one hand, is a little unsteady while walking, has slight loss of vision in one eye, and often needs to urinate urgently. | * |  | ○ |  |  |  |  | ○ |  |  |  |  | ○ |
| 65 | GBD 2010 original | Multiple sclerosis, moderate | needs help walking, has difficulty with writing and arm coordination, has loss of vision in one eye and cannot control urinating. | * |  | ○ |  |  | ○ |  |  |  |  |  |  | ○ |
| 66 | GBD 2010 original | Multiple sclerosis, severe | has slurred speech and difficulty swallowing. The person has weak arms and hands, very limited and stiff leg movement, has loss of vision in both eyes and cannot control urinating. | * |  | ○ |  |  | ○ |  | ○ |  |  |  |  | ○ |
| 67 | European original | Epilepsy, seizures >= once a month | has sudden seizures one or more times each month, with violent muscle contractions and stiffness, loss of consciousness, and loss of urine or bowel control. Between seizures the person has memory loss and difficulty concentrating. | * |  |  |  |  |  |  |  |  |  |  | ○ | ○ |
| 68 | European original | Epilepsy, seizures < once a month | has sudden seizures two to five times a year, with violent muscle contractions and stiffness, loss of consciousness, and loss of urine or bowel control. | * |  |  |  |  |  |  |  |  |  |  |  |  |
| 69 | GBD 2010 original | Parkinson's disease, mild | has mild tremors and moves a little slowly, but is able to walk and do daily activities without assistance. | * |  | ○ |  |  |  |  |  |  |  |  |  |  |
| 70 | GBD 2010 original | Parkinson's disease, moderate | has moderate tremors and moves slowly, which causes some difficulty in walking and daily activities. The person has some trouble swallowing, talking, sleeping, and remembering things. | * |  | ○ |  |  |  |  |  |  |  | ○ | ○ | ○ |
| 71 | GBD 2010 original | Parkinson's disease, severe | has severe tremors and moves very slowly, which causes great difficulty in walking and daily activities. The person falls easily and has a lot of difficulty talking, swallowing, sleeping, and remembering things. | * |  | ○ |  |  |  |  |  |  |  | ○ | ○ | ○ |
| Mental, behavioural, and substance use disorders | | |  |  |  |  |  |  |  |  |  |  |  |  |  |  |
| 72 | European original | Alcohol use disorder, very mild | drinks alcohol daily and has difficulty controlling the urge to drink. When sober, the person functions normally. | * |  |  |  |  |  |  |  |  | ○ |  |  |  |
| 73 | GBD 2010 original | Alcohol use disorder, mild | drinks a lot of alcohol and sometimes has difficulty controlling the urge to drink. While intoxicated, the person has difficulty performing daily activities. | * |  |  |  |  |  |  |  |  | ○ | ○ |  |  |
| 74 | GBD 2010 original | Alcohol use disorder, moderate | drinks a lot, gets drunk almost every week and has great difficulty controlling the urge to drink. Drinking and recovering cause great difficulty in daily activities, sleep loss, and fatigue. | * |  |  |  |  | ○ |  |  |  | ○ | ○ |  | ○ |
| 75 | GBD 2010 original | Alcohol use disorder, severe | gets drunk almost every day and is unable to control the urge to drink. Drinking and recovering replace most daily activities. The person has difficulty thinking, remembering and communicating, and feels constant pain and fatigue. | * |  |  | ○ |  | ○ |  |  |  | ○ |  | ○ |  |
| 76 | Japanese new | Drug dependence, mild | uses unlegal drugs at least once a week and has some difficulty controlling the habit. When not using, the person functions normally. | * |  |  |  |  |  |  |  |  | ○ |  |  |  |
| 77 | Japanese new | Drug dependence | uses unlegal drugs daily and has difficulty controlling the habit. The person sometimes has mood swings, anxiety and hallucinations, and has some difficulty in daily activities. | * |  |  |  | ○ |  |  |  |  | ○ | ○ | ○ |  |
| 78 | GBD 2010 original | Anxiety disorders, mild | feels mildly anxious and worried, which makes it slightly difficult to concentrate, remember things, and sleep. The person tires easily but is able to perform daily activities. | * | * |  |  | ○ | ○ |  |  |  |  |  | ○ |  |
| 79 | GBD 2010 original | Anxiety disorders, moderate | feels anxious and worried, which makes it difficult to concentrate, remember things, and sleep. The person tires easily and finds it difficult to perform daily activities. | * | * |  |  | ○ | ○ |  |  |  |  | ○ | ○ |  |
| 80 | GBD 2010 original | Anxiety disorders, severe | constantly feels very anxious and worried, which makes it difficult to concentrate, remember things and sleep. The person has lost pleasure in life and thinks about suicide. | * | * |  |  | ○ | ○ |  |  |  |  | ○ | ○ |  |
| 81 | GBD 2010 modified | Major depressive disorder, mild episode | feels persistent sadness and has lost interest in usual activities. The person sometimes sleeps badly, feels tired, or has trouble concentrating but still manages to function in daily life with extra effort. | * | * |  |  | ○ | ○ |  |  |  |  |  |  | ○ |
| 82 | GBD 2010 original | Major depressive disorder, moderate episode | has constant sadness and has lost interest in usual activities. The person has some difficulty in daily life, sleeps badly, has trouble concentrating, and sometimes thinks about harming himself (or herself). | * | * |  |  | ○ | ○ |  |  |  |  | ○ |  | ○ |
| 83 | GBD 2010 original | Major depressive disorder, severe episode | has overwhelming, constant sadness and cannot function in daily life. The person sometimes loses touch with reality and wants to harm or kill himself (or herself). | * | * |  |  | ○ | ○ |  |  |  |  | ○ |  | ○ |
| 84 | GBD 2010 original | Bipolar disorder: manic episode | is hyperactive, hears and believes things that are not real, and engages in impulsive and aggressive behavior that endanger the person and others. | * | * |  |  | ○ |  |  |  |  |  |  | ○ |  |
| 85 | GBD 2010 original | Bipolar disorder: residual state | has mild mood swings, irritability and some difficulty with daily activities. | * | * |  |  | ○ |  |  |  |  |  | ○ |  |  |
| 86 | GBD 2010 original | Schizophrenia: acute state | hears and sees things that are not real and is afraid, confused, and sometimes violent. The person has great difficulty with communication and daily activities, and sometimes wants to harm or kill himself (or herself). | * |  |  |  | ○ |  |  |  |  |  | ○ | ○ |  |
| 87 | GBD 2010 original | Schizophrenia: residual state | hears and sees things that are not real and has trouble communicating. The person can be forgetful, has difficulty with daily activities, and thinks about hurting himself (or herself). | * |  |  |  | ○ |  |  |  |  |  | ○ | ○ |  |
| 88 | GBD 2010 original | Anorexia nervosa | feels an overwhelming need to starve and exercises excessively to lose weight. The person is very thin, weak and anxious. | * | * |  |  | ○ | ○ |  |  |  |  |  |  |  |
| 89 | GBD 2010 original | Bulimia nervosa | has uncontrolled overeating followed by guilt, starving, and vomiting to lose weight. | * | * |  |  | ○ |  |  |  |  |  |  |  |  |
| 90 | GBD 2010 original | Attention deficit hyperactivity disorder | is hyperactive and has difficulty concentrating, remembering things, and completing tasks. | * |  |  |  | ○ |  |  |  |  |  | ○ | ○ |  |
| 91 | GBD 2010 original | Conduct disorder | has frequent behavior problems, which are sometimes violent. The person often has difficulty interacting with other people and feels irritable. | * |  |  |  | ○ |  |  |  |  |  | ○ |  |  |
| 92 | GBD 2010 original | Asperger's syndrome | has difficulty interacting with other people, and is slow to understand or respond to questions. The person is often preoccupied with one thing and has some difficulty with basic daily activities. | * |  |  |  | ○ |  |  |  |  |  |  | ○ |  |
| 93 | GBD 2010 original | Autism | has severe problems interacting with others and difficulty understanding simple questions or directions. The person has great difficulty with basic daily activities and becomes distressed by any change in routine. | * |  |  |  | ○ |  |  |  |  |  |  | ○ |  |
| 94 | GBD 2010 modified | Intellectual disability, borderline | is slow in learning at school. As an adult, the person has some difficulty doing complex or unfamiliar tasks but otherwise functions independently. | * |  |  |  |  |  |  |  |  |  | ○ | ○ |  |
| 95 | GBD 2010 modified | Intellectual disability, mild | has low intelligence and is slow in learning at school. As an adult, the person can live independently, but often needs help to raise children and can only work at simple supervised jobs. | * |  |  |  |  |  |  |  |  |  | ○ | ○ |  |
| 96 | GBD 2010 modified | Intellectual disability, moderate | has low intelligence, and is slow in learning to speak and to do even simple tasks. As an adult, the person requires a lot of support to live independently and raise children. The person can only work at the simplest supervised jobs. | * |  |  |  |  |  |  |  |  |  | ○ | ○ |  |
| 97 | GBD 2010 modified | Intellectual disability, severe | has very low intelligence and cannot speak more than a few words, needs constant supervision and help with most daily activities, and can do only the simplest tasks. | * |  |  |  |  |  |  |  |  |  | ○ | ○ |  |
| 98 | GBD 2010 modified | Intellectual disability, profound | has very low intelligence, has almost no language, and does not understand even the most basic requests or instructions. The person requires constant supervision and help for all activities. | * |  |  |  |  |  |  |  |  |  | ○ | ○ |  |
| Hearing and vision loss | | |  |  |  |  |  |  |  |  |  |  |  |  |  |  |
| 99 | GBD 2010 modified | Hearing loss, mild | has great difficulty hearing and understanding another person talking in a noisy place (for example, on an urban street). | * |  |  |  |  |  |  | ○ |  |  |  |  |  |
| 100 | GBD 2010 modified | Hearing loss, moderate | is unable to hear and understand another person talking in a noisy place (for example, on an urban street), and has difficulty hearing another person talking even in a quiet place or on the phone. | * |  |  |  |  |  |  | ○ |  |  |  |  |  |
| 101 | GBD 2010 modified | Hearing loss, severe | is unable to hear and understand another person talking, even in a quiet place, and unable to take part in a phone conversation. Difficulties with communicating and relating to others cause emotional impact at times (for example worry or depression). | * |  |  |  | ○ |  |  | ○ |  |  | ○ |  |  |
| 102 | GBD 2010 modified | Hearing loss, profound | is unable to hear and understand another person talking, even in a quiet place, is unable to take part in a phone conversation, and has great difficulty hearing anything in any other situation. Difficulties with communicating and relating to others often cause worry, depression or loneliness. | * |  |  |  | ○ |  |  | ○ |  |  | ○ |  |  |
| 103 | GBD 2010 modified | Hearing loss, complete | cannot hear at all in any situation, including even the loudest sounds, and cannot communicate verbally or use a phone. Difficulties with communicating and relating to others often cause worry, depression or loneliness. | * |  |  |  | ○ |  |  | ○ |  |  | ○ |  |  |
| 104 | GBD 2010 modified | Hearing loss, mild, with ringing | has great difficulty hearing and understanding another person talking in a noisy place (for example, on an urban street), and sometimes has annoying ringing in the ears. | * | * |  |  |  |  |  | ○ |  |  |  |  |  |
| 105 | GBD 2010 modified | Hearing loss, moderate, with ringing | is unable to hear and understand another person talking in a noisy place (for example, on an urban street), has difficulty hearing another person talking even in a quiet place or on the phone, and has annoying ringing in the ears for 5 minutes at a time, almost every day. | * | * |  |  |  |  |  | ○ |  |  |  |  |  |
| 106 | GBD 2010 modified | Hearing loss, severe, with ringing | is unable to hear and understand another person talking, even in a quiet place, is unable to take part in a phone conversation, and has annoying ringing in the ears for more than 5 minutes at a time, almost every day. Difficulties with communicating and relating to others cause emotional impact at times (for example worry or depression). | * | * |  |  | ○ |  |  | ○ |  |  | ○ |  |  |
| 107 | GBD 2010 modified | Hearing loss, profound, with ringing | is unable to hear and understand another person talking, even in a quiet place, is unable to take part in a phone conversation, has great difficulty hearing anything in any other situation, and has annoying ringing in the ears for more than 5 minutes at a time, several times a day. Difficulties with communicating and relating to others often cause worry, depression, or loneliness. | * | * |  |  | ○ |  |  | ○ |  |  | ○ |  |  |
| 108 | GBD 2010 modified | Hearing loss, complete, with ringing | cannot hear at all in any situation, including even the loudest sounds, and cannot communicate verbally or use a phone, and has very annoying ringing in the ears for more than half of the day. Difficulties with communicating and relating to others often cause worry, depression or loneliness. | * | * |  |  | ○ |  |  | ○ |  |  | ○ |  |  |
| 109 | GBD 2010 original | Distance vision, mild impairment | has some difficulty with distance vision, for example reading signs, but no other problems with eyesight. | * |  |  |  |  |  |  | ○ |  |  |  |  |  |
| 110 | GBD 2010 original | Distance vision, moderate impairment | has vision problems that make it difficult to recognize faces or objects across a room. | * |  |  |  |  |  |  | ○ |  |  |  |  |  |
| 111 | GBD 2010 original | Distance vision, severe impairment | has severe vision loss, which causes difficulty in daily activities, some emotional impact (for example worry), and some difficulty going outside the home without assistance. | * |  |  |  | ○ |  |  | ○ |  |  | ○ |  |  |
| 112 | GBD 2010 modified | Distance vision blindness | is completely blind, which causes great difficulty in some daily activities, and in going outside the home without assistance. The person sometimes feels worried or depressed due to social isolation. | * |  |  |  | ○ |  |  | ○ |  |  | ○ |  |  |
| 113 | GBD 2010 original | Near vision impairment | has difficulty seeing things that are nearer than 3 feet, but has no difficulty with seeing things at a distance. | * |  |  |  |  |  |  | ○ |  |  |  |  |  |
| Musculoskeletal disorders | | |  |  |  |  |  |  |  |  |  |  |  |  |  |  |
| 114 | European original | Low back pain, mild | has mild back pain, which causes some difficulty dressing, standing, and lifting things. | * | * |  | ○ |  |  |  |  |  |  | ○ |  |  |
| 115 | European original | Low back pain, moderate | has moderate back pain, which causes difficulty dressing, sitting, standing, walking, and lifting things. | * | * |  | ○ |  |  |  |  |  |  | ○ |  |  |
| 116 | GBD 2010 original | Back pain, severe, without leg pain | has severe back pain, which causes difficulty dressing, sitting, standing, walking, and lifting things. The person sleeps poorly and feels worried. | * | * |  | ○ | ○ |  |  |  |  |  | ○ |  | ○ |
| 117 | GBD 2010 original | Back pain, severe, with leg pain | has severe back and leg pain, which causes difficulty dressing, sitting, standing, walking, and lifting things. The person sleeps poorly and feels worried. | * | * |  | ○ | ○ |  |  |  |  |  | ○ |  | ○ |
| 118 | GBD 2010 original | Back pain, most severe, without leg pain | has constant back pain, which causes difficulty dressing, sitting, standing, walking, and lifting things. The person sleeps poorly, is worried, and has lost some enjoyment in life. | * | * |  | ○ | ○ |  |  |  |  |  | ○ |  | ○ |
| 119 | GBD 2010 original | Back pain, most severe, with leg pain | has constant back and leg pain, which causes difficulty dressing, sitting, standing, walking, and lifting things. The person sleeps poorly, is worried, and has lost some enjoyment in life. | * | * |  | ○ | ○ |  |  |  |  |  | ○ |  | ○ |
| 120 | GBD 2010 original | Neck pain, mild | has neck pain, and has difficulty turning the head and lifting things. | * | * |  | ○ |  |  |  |  |  |  |  |  |  |
| 121 | GBD 2010 original | Neck pain, moderate | has constant neck pain, and has difficulty turning the head, holding arms up, and lifting things | * | * |  | ○ |  |  |  |  |  |  | ○ |  |  |
| 122 | GBD 2010 original | Neck pain, severe | has severe neck pain, and difficulty turning the head and lifting things. The person gets headaches and arm pain, sleeps poorly, and feels tired and worried. | * | * |  | ○ | ○ |  |  |  |  |  | ○ |  | ○ |
| 123 | GBD 2010 original | Neck pain, most severe | has constant neck pain and arm pain, and difficulty turning the head, holding arms up, and lifting things. The person gets headaches, sleeps poorly, and feels tired and worried. | * | * |  | ○ | ○ |  |  |  |  |  | ○ |  | ○ |
| 124 | GBD 2010 original | Musculoskeletal problems, lower limbs, mild | has pain in the leg, which causes some difficulty running, walking long distances, and getting up and down. | * | * | ○ | ○ |  |  |  |  |  |  |  |  |  |
| 125 | GBD 2010 original | Musculoskeletal problems, lower limbs, moderate | has moderate pain in the leg, which makes the person limp, and causes some difficulty walking, standing, lifting and carrying heavy things, getting up and down and sleeping. | * | * | ○ | ○ |  |  |  |  |  |  |  |  | ○ |
| 126 | GBD 2010 original | Musculoskeletal problems, lower limbs, severe | has severe pain in the leg, which makes the person limp and causes a lot of difficulty walking, standing, lifting and carrying heavy things, getting up and down, and sleeping. | * | * | ○ | ○ |  |  |  |  |  |  |  |  | ○ |
| 127 | GBD 2010 original | Musculoskeletal problems, upper limbs, mild | has mild pain and stiffness in the arms and hands. The person has some difficulty lifting, carrying and holding things. | * | * |  | ○ |  |  |  |  |  |  | ○ |  |  |
| 128 | GBD 2010 original | Musculoskeletal problems, upper limbs, moderate | has moderate pain and stiffness in the arms and hands, which causes difficulty lifting, carrying, and holding things, and trouble sleeping because of the pain. | * | * |  | ○ |  |  |  |  |  |  | ○ |  | ○ |
| 129 | GBD 2010 original | Musculoskeletal problems, generalized, moderate | has pain and deformity in most joints, causing difficulty moving around, getting up and down, and using the hands for lifting and carrying. The person often feels fatigue. | * | * | ○ | ○ |  | ○ |  |  |  |  |  |  |  |
| 130 | GBD 2010 original | Musculoskeletal problems, generalized, severe | has severe, constant pain and deformity in most joints, causing difficulty moving around, getting up and down, eating, dressing, lifting, carrying and using the hands. The person often feels sadness, anxiety and extreme fatigue. | * | * | ○ | ○ | ○ |  | ○ |  |  |  | ○ |  |  |
| 131 | European original | Grout: Acute | has severe pain and swelling in the leg, making it very difficult to get up and down, stand, walk, lift, and carry heavy things. The person has trouble sleeping because of the pain. |  | * | ○ | ○ |  |  |  |  |  |  |  |  | ○ |
| Injury | | |  |  |  |  |  |  |  |  |  |  |  |  |  |  |
| 132 | GBD 2010 modified | Amputation of finger(s), excluding thumb | has lost a finger of one hand. At times there is pain and tingling in the stump. | * |  |  | ○ |  |  | ○ |  |  |  |  |  |  |
| 133 | GBD 2010 original | Amputation of thumb (long term) | has lost one thumb, causing some difficulty in using the hand, pain, and tingling in the stump. | * |  |  | ○ |  |  | ○ |  |  |  |  |  |  |
| 134 | GBD 2010 modified | Amputation of one upper limb (with treatment) | has lost one hand and part of the arm, leaving pain and tingling in the stump. The person has an artificial arm that makes it possible to lift objects and do daily activities such as cooking, with some extra effort. | * |  |  | ○ |  |  | ○ |  |  |  | ○ |  |  |
| 135 | European original | Amputation of one upper limb (long term, without treatment) | has lost one hand and part of the arm, leaving pain and tingling in the stump. The person needs help from others to lift objects or do daily activities such as cooking. | * |  |  | ○ |  |  | ○ |  |  |  | ○ |  |  |
| 136 | GBD 2010 modified | Amputation of both upper limbs (long term, with treatment) | has lost part of both arms, leaving pain and tingling in the stumps. The person has two artificial arms that make it possible to do daily activities, with a great deal of extra effort. | * |  |  | ○ |  |  | ○ |  |  |  | ○ |  |  |
| 137 | GBD 2010 modified | Amputation of both upper limbs (long term, without treatment) | has lost part of both arms, leaving pain and tingling in the stumps. The person needs a great deal of help from others to do even basic daily activities such as eating and using the toilet, and the person is very limited in other activities. | * |  |  | ○ |  |  | ○ |  |  |  | ○ |  |  |
| 138 | GBD 2010 original | Amputation of toe | has lost one toe, leaving occasional pain and tingling in the stump. | * |  |  | ○ |  |  | ○ |  |  |  |  |  |  |
| 139 | GBD 2010 modified | Amputation of one lower limb (long term, with treatment) | has lost part of one leg, leaving pain and tingling in the stump. The person has an artificial leg that helps in moving around. | * |  | ○ | ○ |  |  | ○ |  |  |  |  |  |  |
| 140 | GBD 2010 original | Amputation of one lower limb (long term, without treatment) | has lost part of one leg, leaving pain and tingling in the stump. The person does not have an artificial leg, has frequent sores, and uses crutches. | * |  | ○ | ○ |  |  | ○ |  |  |  |  |  |  |
| 141 | GBD 2010 modified | Amputation of both lower limbs (long term, with treatment) | has lost part of both legs, leaving pain and tingling in the stumps. The person has two artificial legs that make moving around possible, with extra effort. | * |  | ○ | ○ |  |  | ○ |  |  |  |  |  |  |
| 142 | GBD 2010 modified | Amputation of both lower limbs (long term, without treatment) | has lost part of both legs, leaving pain, tingling, and frequent sores in the stumps. The person has great difficulty moving around, has episodes of depression and anxiety, and needs help from others to do many daily activities. | * |  | ○ | ○ | ○ |  | ○ |  |  |  | ○ |  |  |
| 143 | GBD 2010 original | Burns, <20% total burned surface area or < 10% total burned surface area if head/neck or hands/wrist involved (long term, with or without treatment) | has scars caused by a burn. The scars are sometimes painful and itchy. | * |  |  | ○ |  |  | ○ |  |  |  |  |  |  |
| 144 | GBD 2010 original | Burns, ≥20% total burned surface area (short term, with or without treatment) | has a painful burn over a large part of the body. Parts of the burned area have lost feeling, and the person feels anxious and unwell. |  | * |  | ○ | ○ |  | ○ |  |  |  |  |  |  |
| 145 | GBD 2010 original | Burns, ≥20% total burned surface area or ≥10% total burned surface area if head/neck or hands/wrist involved (long term, with treatment) | has scars caused by burns over a large part of the body. The scars are frequently painful and itchy, and the person is often sad. | * |  |  | ○ | ○ |  | ○ |  |  |  |  |  |  |
| 146 | GBD 2010 original | Burns, ≥20% total burned surface area or ≥10% total burned surface area if head/neck or hands/wrist involved (long term, without treatment) | has severe, disfiguring and itchy scars caused by burns over a large part of the body. The person cannot move some joints, feels sad, and has great difficulty with self-care such as dressing and toileting. | * |  |  | ○ | ○ |  | ○ |  |  |  | ○ |  |  |
| 147 | GBD 2010 original | Crush injury (short or long term, with or without treatment) | had part of the body crushed, leaving pain, swelling, tingling and limited feeling in the affected area. | * | * |  | ○ |  |  |  |  |  |  |  |  |  |
| 148 | GBD 2010 original | Dislocation of hip (long term, with or without treatment) | walks with a limp and feels discomfort when walking. | * | * | ○ | ○ |  |  |  |  |  |  |  |  |  |
| 149 | GBD 2010 original | Dislocation of knee (long term, with or without treatment) | has a knee out of joint, causing pain and difficulty moving the knee, which sometimes gives way. The person needs crutches for walking and help with self-care such as dressing. | * | * | ○ | ○ |  |  |  |  |  |  | ○ |  |  |
| 150 | GBD 2010 original | Dislocation of shoulder (long term, with or without treatment) | has a shoulder that is out of joint, causing pain and difficulty moving. The person has difficulty with daily activities such as dressing and cooking. | * | * |  | ○ |  |  |  |  |  |  | ○ |  |  |
| 151 | GBD 2010 original | Other injuries of muscle and tendon (includes sprains, strains and dislocations other than shoulder, knee, hip) | has a strained muscle that causes pain and swelling. | * | * |  | ○ |  |  |  |  |  |  |  |  |  |
| 152 | GBD 2010 original | Drowning and nonfatal submersion (short or long term, with or without treatment) | has breathlessness, anxiety, cough, and vomiting. |  | * |  |  |  |  |  |  |  |  |  |  | ○ |
| 153 | GBD 2010 original | Fracture of clavicle, scapula or humerus (short or long term, with or without treatment) | has a broken shoulder bone, which is painful and swollen. The person cannot use the affected arm and has difficulty with getting dressed. |  | * |  | ○ |  |  |  |  |  |  | ○ |  |  |
| 154 | GBD 2010 modified | Fracture of face bone (short or long term with or without treatment) | has a broken cheek bone or a broken nose or chipped teeth, with swelling and severe pain. |  | * |  | ○ |  |  | ○ |  |  |  |  |  |  |
| 155 | GBD 2010 original | Fracture of foot bones (short term, with or without treatment) | has a broken foot bone, which causes pain, swelling, and difficulty walking. |  | * | ○ | ○ |  |  |  |  |  |  |  |  |  |
| 156 | GBD 2010 original | Fracture of foot bones (long term, without treatment) | had a broken foot in the past that did not heal properly. The person now has pain in the foot and has some difficulty walking. | * |  | ○ | ○ |  |  |  |  |  |  |  |  |  |
| 157 | GBD 2010 original | Fracture of hand (short term, with or without treatment) | has a broken hand, causing pain and swelling. |  | * | ○ | ○ |  |  |  |  |  |  |  |  |  |
| 158 | GBD 2010 original | Fracture of hand (long term, without treatment) | has stiffness in the hand and a weak grip. | * |  |  |  |  |  |  |  |  |  | ○ |  |  |
| 159 | GBD 2010 original | Fracture of neck of femur (short term, with or without treatment) | has broken a hip and is in pain. The person cannot stand or walk, and needs help washing, dressing, and going to the toilet. |  | * | ○ | ○ |  |  |  |  |  |  | ○ |  |  |
| 160 | GBD 2010 original | Fracture of neck of femur (long term, with treatment) | had a broken hip in the past, which was fixed with treatment. The person can only walk short distances, has discomfort when moving around, and has some difficulty in daily activities. | * |  | ○ | ○ |  |  |  |  |  |  | ○ |  |  |
| 161 | GBD 2010 original | Fracture of neck of femur (long term, without treatment) | had a broken hip bone in the past, which was never treated and did not heal properly. The person cannot get out of bed and needs help washing and going to the toilet. | * |  | ○ |  |  |  |  |  |  |  | ○ |  |  |
| 162 | GBD 2010 original | Fracture, other than femoral neck (short term, with or without treatment) | has a broken thigh bone. The person has severe pain and swelling and cannot walk. |  | * | ○ | ○ |  |  |  |  |  |  |  |  |  |
| 163 | GBD 2010 original | Fracture, other than femoral neck (long term, without treatment) | had a broken thigh bone in the past, which was never treated and did not heal properly. The person now has a limp and discomfort when walking. | * |  | ○ | ○ |  |  |  |  |  |  |  |  |  |
| 164 | GBD 2010 original | Fracture of patella, tibia or fibula or ankle (short term, with or without treatment) | has a broken shin bone, which causes severe pain, swelling, and difficulty walking. |  | * | ○ | ○ |  |  |  |  |  |  |  |  |  |
| 165 | GBD 2010 original | Fracture of patella, tibia or fibula or ankle (long term, with or without treatment) | had a broken shin bone in the past that did not heal properly. The person has pain in the knee and ankle, and has difficulty walking. | * |  | ○ | ○ |  |  |  |  |  |  |  |  |  |
| 166 | GBD 2010 original | Fracture of pelvis (short term) | has a broken pelvis bone, with swelling and bruising. The person has severe pain, and cannot walk or do daily activities. |  | * | ○ | ○ |  |  |  |  |  |  |  |  |  |
| 167 | GBD 2010 original | Fracture of pelvis (long term) | had a broken pelvis in the past and now walks with a limp. There is often pain in the back and groin, and when urinating and sitting for a long time. | * |  |  | ○ |  |  |  |  |  |  |  |  | ○ |
| 168 | GBD 2010 original | Fracture of radius or ulna (short term, with or without treatment) | has a broken forearm, which causes severe pain, swelling, and limited movement. |  | * |  | ○ |  |  |  |  |  |  |  |  |  |
| 169 | GBD 2010 original | Fracture of radius or ulna (long term, without treatment) | had a broken forearm in the past that did not heal properly, causing some pain and limited movement in the elbow and wrist. The person has difficulty with daily activities such as dressing. | * |  |  | ○ |  |  |  |  |  |  | ○ |  |  |
| 170 | GBD 2010 original | Fracture of skull (short or long term, with or without treatment) | has a broken skull, but does not have brain damage. The broken area is painful and swollen. |  | * |  | ○ |  |  |  |  |  |  |  |  |  |
| 171 | GBD 2010 original | Fracture of sternum and/or fracture of one or two ribs (short term, with or without treatment) | has a broken rib that causes severe pain in the chest, especially when breathing in. The person has difficulty with daily activities such as dressing. |  | * |  | ○ |  |  |  |  |  |  | ○ |  |  |
| 172 | GBD 2010 original | Fracture of vertebral column (short or long term, with or without treatment) | has broken back bones and is in pain, but still has full use of arms and legs. |  | * |  | ○ |  |  |  |  |  |  |  |  |  |
| 173 | GBD 2010 original | Fractures, treated (long term) | has slight pain in a bone that was broken in the past. | * |  |  | ○ |  |  |  |  |  |  |  |  |  |
| 174 | GBD 2010 original | Injured nerves (short term) | has a nerve injury, which causes difficulty moving and some loss of feeling in the affected area. |  | * |  |  |  |  |  |  |  |  |  |  | ○ |
| 175 | GBD 2010 original | Injured nerves (long term) | had a nerve injury in the past, which continues to cause some difficulty moving. The person often injures the affected part because it is numb. | * |  |  |  |  |  |  |  |  |  |  |  | ○ |
| 176 | GBD 2010 original | Injury to eyes (short term) | has an injury to one eye, which causes pain and difficulty seeing. |  | * |  | ○ |  |  |  | ○ |  |  |  |  |  |
| 177 | European original | Concussion | has headaches, dizziness, nausea and difficulty concentrating. |  | * |  | ○ |  |  |  |  |  |  |  | ○ | ○ |
| 178 | GBD 2010 original | Severe traumatic brain injury, short term (with or without treatment) | cannot concentrate and has headaches, memory problems, dizziness, and feels angry. |  | * |  | ○ | ○ |  |  |  |  |  |  | ○ | ○ |
| 179 | GBD 2010 original | Traumatic brain injury, long-term consequences, minor (with or without treatment) | has episodes of headaches, memory problems, and difficulty concentrating. | * |  |  | ○ | ○ |  |  |  |  |  |  | ○ |  |
| 180 | GBD 2010 original | Traumatic brain injury, long-term consequences, moderate (with or without treatment) | has frequent headaches, memory problems, difficulty concentrating, and dizziness. The person is often anxious and moody. | * |  |  | ○ | ○ |  |  |  |  |  |  | ○ |  |
| 181 | GBD 2010 original | Traumatic brain injury, long-term consequences, severe (with or without treatment) | cannot think clearly and has frequent headaches, memory problems, difficulty concentrating and dizziness. The person is often anxious and moody, and depends on others for feeding, toileting, dressing and walking. | * |  |  | ○ | ○ |  |  |  |  |  |  | ○ |  |
| 182 | GBD 2010 original | Open wound (short term, with or without treatment) | has a cut in the skin, which causes pain and numbness around the cut. |  | * |  | ○ |  |  |  |  |  |  |  |  |  |
| 183 | GBD 2010 original | Poisoning (short term with or without treatment) | has drowsiness, stomach pain and vomiting. |  | * |  | ○ |  |  |  |  |  |  |  |  | ○ |
| 184 | GBD 2010 original | Severe chest injury (long term, with or without treatment) | had a severe chest injury in the past that has now healed. The person still gets breathless when walking and feels discomfort in the chest. | * |  |  | ○ |  |  |  |  |  |  |  |  | ○ |
| 185 | GBD 2010 original | Severe chest injury (short term, with or without treatment) | has a serious chest injury, which causes severe pain, shortness of breath and anxiety. |  | * |  | ○ |  |  |  |  |  |  |  |  | ○ |
| 186 | GBD 2010 modified | Spinal cord lesion below neck level (treated) | is paralyzed from the waist down, cannot feel or move the legs and has difficulties with urine and bowel control. The person uses a wheelchair to move around. | * |  | ○ |  |  |  |  |  |  |  |  |  | ○ |
| 187 | GBD 2010 modified | Spinal cord lesion below neck level (untreated) | is paralyzed from the waist down, cannot feel or move the legs and has difficulties with urine and bowel control. Legs are in fixed, bent positions, and the person gets frequent infections and pressure sores. | * |  | ○ | ○ |  |  |  |  | ○ |  |  |  | ○ |
| 188 | GBD 2010 modified | Spinal cord lesion at neck level (treated) | is paralyzed from the neck down, with no feeling or control over any part of the body below the neck, and no urine or bowel control. | * |  | ○ | ○ |  |  |  |  |  |  |  |  | ○ |
| 189 | GBD 2010 modified | Spinal cord lesion at neck level (untreated) | is paralyzed from the neck down, with no feeling or control over any part of the body below the neck, and no urine or bowel control. Arms and legs are in fixed, bent positions, and the person gets frequent infections and pressure sores. | * |  | ○ | ○ |  |  |  |  | ○ |  |  |  | ○ |
| Other | | |  |  |  |  |  |  |  |  |  |  |  |  |  |  |
| 190 | GBD 2010 original | Abdominopelvic problem, mild | has some pain in the belly that causes nausea but does not interfere with daily activities. | * | * |  | ○ |  |  |  |  |  |  |  |  | ○ |
| 191 | GBD 2010 original | Abdominopelvic problem, moderate | has pain in the belly and feels nauseous. The person has difficulties with daily activities. | * | * |  | ○ |  |  |  |  |  |  | ○ |  | ○ |
| 192 | GBD 2010 original | Abdominopelvic problem, severe | has severe pain in the belly and feels nauseous. The person is anxious and unable to carry out daily activities. | * | * |  |  |  | ○ |  |  |  |  |  |  |  |
| 193 | GBD 2010 original | Anemia, mild | feels slightly tired and weak at times, but this does not interfere with normal daily activities. | * | * |  |  |  | ○ |  |  |  |  | ○ |  |  |
| 194 | GBD 2010 original | Anemia, moderate | feels moderate fatigue, weakness, and shortness of breath after exercise, making daily activities more difficult. | * | * |  |  |  | ○ |  |  |  |  | ○ |  |  |
| 195 | GBD 2010 original | Anemia, severe | feels very weak, tired and short of breath, and has problems with activities that require physical effort or deep concentration. | * | * |  |  |  |  |  |  |  |  |  |  |  |
| 196 | GBD 2010 original | Periodontitis | has minor bleeding of the gums from time to time, with mild discomfort. | * | * |  | ○ |  |  |  |  |  |  |  |  | ○ |
| 197 | GBD 2010 original | Dental caries: symptomatic | has a toothache, which causes some difficulty in eating. | * | * |  | ○ |  |  |  |  |  |  |  |  |  |
| 198 | GBD 2010 original | Severe tooth loss | has lost more than 20 teeth including front and back, and has great difficulty in eating meat, fruits, and vegetables. | * |  |  |  |  |  |  |  |  |  | ○ |  |  |
| 199 | GBD 2010 original | Disfigurement: level 1 | has a slight, visible physical deformity that others notice, which causes some worry and discomfort. | * |  |  |  |  |  | ○ |  |  |  |  |  |  |
| 200 | GBD 2010 original | Disfigurement: level 2 | has a visible physical deformity that causes others to stare and comment. As a result, the person is worried and has trouble sleeping and concentrating. | * |  |  |  | ○ |  | ○ |  |  |  |  |  | ○ |
| 201 | GBD 2010 original | Disfigurement: level 3 | has an obvious physical deformity that makes others uncomfortable, which causes the person to avoid social contact, feel worried, sleep poorly, and think about suicide. | * |  |  |  | ○ |  | ○ |  |  |  |  |  | ○ |
| 202 | GBD 2010 original | Generic uncomplicated disease: worry and daily medication | has a chronic disease that requires medication every day and causes some worry but minimal interference with daily activities. | * | * |  |  | ○ |  |  |  |  |  |  |  |  |
| 203 | GBD 2010 modified | Generic uncomplicated disease: anxiety about diagnosis | has a disease diagnosis that causes some worry but minimal interference with daily activities. | * | * |  |  | ○ |  |  |  |  |  |  |  |  |
| 204 | GBD 2010 original | Severe wasting | is extremely skinny and has no energy. | * |  |  |  |  | ○ |  |  |  |  |  |  | ○ |
| 205 | GBD 2010 original | Speech problems | has difficulty speaking, and others find it difficult to understand. | * |  |  |  |  |  |  |  |  |  |  |  | ○ |
| 206 | GBD 2010 original | Motor impairment, mild | has some difficulty in moving around but is able to walk without help. | * | * | ○ |  |  |  |  |  |  |  |  |  |  |
| 207 | GBD 2010 original | Motor impairment, moderate | has some difficulty in moving around, and difficulty in lifting and holding objects, dressing and sitting upright, but is able to walk without help. | * | * | ○ |  |  |  |  |  |  |  | ○ |  |  |
| 208 | GBD 2010 original | Motor impairment, severe | is unable to move around without help, and is not able to lift or hold objects, get dressed or sit upright. | * | * | ○ |  |  |  |  |  |  |  | ○ |  |  |
| 209 | GBD 2010 modified | Motor plus cognitive impairments, mild | has some difficulty in moving around but is able to walk without help. The person is slow in learning at school. As an adult, the person has some difficulty doing complex or unfamiliar tasks but otherwise functions independently. | * |  | ○ |  |  |  |  |  |  |  |  | ○ |  |
| 210 | GBD 2010 modified | Motor plus cognitive impairments, moderate | has some difficulty in moving around, holding objects, dressing and sitting upright, but can walk without help. The person has low intelligence and is slow in learning to speak and to do simple tasks. As an adult, the person requires support to live independently and raise children and can only work at simple supervised jobs. | * |  | ○ |  |  |  |  |  |  |  | ○ | ○ |  |
| 211 | GBD 2010 modified | Motor plus cognitive impairments, severe | cannot move around without help, and cannot lift or hold objects, get dressed or sit upright. The person also has very low intelligence, speaks few words, and needs constant supervision and help with all daily activities. | * |  | ○ |  |  |  |  |  |  |  | ○ | ○ |  |
| 212 | European original | Thrombocytopenic purpura | easily bruises and sometimes bleeds from the gums and nose; feels weak and has some difficulty with daily activities. | * | * |  |  |  | ○ |  |  |  |  | ○ |  | ○ |
| 213 | European original | Hypothyroidism | has low energy and feels cold. | * | * |  |  |  | ○ |  |  |  |  |  |  | ○ |
| 214 | European original | Hyperthyroidism | feels nervous, has palpitations, sweats a lot and has difficulty sleeping. | * | * |  |  | ○ |  |  |  |  |  |  |  | ○ |
| 215 | European original | Vertigo and balance disorder (Menière, labyrinthitis) | has short spells of dizziness and loss of balance; between spells the person is worried the spells will occur again. | * | * |  |  | ○ |  |  |  |  |  |  |  | ○ |
| 216 | European original | Allergic rhinitis (hay fever) | has an itchy, runny nose and frequent sneezing. | * | * |  |  |  |  |  |  |  |  |  |  | ○ |
| 217 | European original | Borderline personality disorder | has unstable moods, often does things without thinking about the consequences and tends to view others and self as either very good or very bad. This leads to troubled relationships and causes the person to feel angry, anxious or depressed. | * | * |  |  | ○ |  |  |  |  |  |  |  |  |
| 218 | European original | Carpal tunnel syndrome | feels pain, numbness or weakness in part of the hand during some activities such as knitting, driving, writing or typing. | * | * |  | ○ |  |  |  |  |  |  | ○ |  |  |
| 219 | European original | Constipation | passes stools infrequently and when it does happen it is painful. | * | * |  | ○ |  |  |  |  |  |  |  |  | ○ |
| 220 | European original | Haemorrhoids | loses some blood when passing stools and at times has pain around the anus. | * | * |  | ○ |  |  |  |  |  |  |  |  | ○ |
| 221 | European original | Heart burn & reflux “GERD” | often has a burning sensation in the back of the chest after eating. | * |  |  | ○ |  |  |  |  |  |  |  |  |  |
| 222 | European original | Insomnia | has difficulty falling or staying asleep. | * | * |  |  |  |  |  |  |  |  |  |  | ○ |
| 223 | European original | Intensive care unit admission | is very ill and often asleep or unconscious; when awake cannot move in bed, cannot speak, is completely dependent on others and is anxious. | * | * |  |  | ○ |  |  |  |  |  | ○ |  | ○ |
| 224 | European original | Invasive device/drain | has a tube inserted in the body to drain fluid, which may cause skin irritation and difficulty in moving around. | * | * | ○ | ○ |  |  |  |  |  |  |  |  |  |
| 225 | European original | Irritable bowel syndrome | has abdominal pain, bloating, flatulence and irregular bowel movements. | * | * |  | ○ |  |  |  |  |  |  |  |  | ○ |
| 226 | European original | Sleep apnoea | feels sleepy during the day and has difficulty concentrating. | * | * |  |  |  |  |  |  |  |  |  |  | ○ |
| 227 | European original | Somatoform disorder | experiences pain, stomach problems or numbness and weakness in part of the body, and feels worry. | * | * |  | ○ | ○ |  |  |  |  |  |  |  |  |
| 228 | European original | Varicose veins | has aches in the legs and swelling of the feet when standing for a long period. | * | * |  | ○ |  |  |  |  |  |  |  |  |  |
| 229 | European original | Trigeminal neuralgia | has episodes of severe pain in the face. | * | * |  | ○ |  |  |  |  |  |  |  |  |  |
| 230 | Japanese new | Vaginal discharge | has a vaginal discharge and feel pain, itchy or swelling in vulva | * | * |  | ○ |  |  |  |  |  |  |  |  | ○ |
| 231 | Japanese new | Dermatitis | has itch, rash or peels of skin and often recurs and feels anxious. Sometimes worries about what others think. | * |  |  |  | ○ |  | ○ |  |  |  |  |  |  |

GBD: Global Burden of Disease study; ADL: activities of daily living; Others: other physical symptoms, including dyspnoea, nausea, palpitations, reduced appetite, sleeping.

**Appendix table 2: Regression analysis results for proportional differences between the Japanese DW and GBD 2013 DW for 226 comparable health states**

| Symptom (number of lay descriptions*) | Coefficient | 95% confidence intervals | P-value |
| --- | --- | --- | --- |
| Mobility (47) | -31.4 | -74.6 to 11.8 | 0.153 |
| Pain (112) | 51.0 | 22.3 to 79.7 | <0.01 |
| Mental symptom (54) | -85.4 | -123.7 to -47.0 | <0.001 |
| Fatigue (40) | -23.8 | -57.9 to 10.2 | 0.169 |
| Disfigurement (23) | 133.0 | -4.5 to 270.6 | 0.058 |
| Sensory symptom (18) | 49.1 | 8.5 to 89.7 | <0.05 |
| Infection/diarrhoea (14) | -6.1 | -107.9 to 95.6 | 0.906 |
| Substance use (4) | -68.6 | -119.0 to -18.1 | <0.01 |
| ADL (86) | -9.7 | -46.3 to 27.0 | 0.603 |
| Cognitive symptom (31) | -23.7 | -49.5 to 2.1 | 0.072 |
| Others (75) | -49.2 | -74.5 to -23.9 | <0.001 |

* The number of lay descriptions add to more than the total because lay descriptions often combine several symptom categories. DW: disability weight; ADL: activities of daily living; Others: other physical symptoms, including dyspnoea, nausea, palpitations, reduced appetite, sleeping problems. A positive coefficient indicates that mention of the symptom in the lay description was associated with a higher value of the Japanese DW than the GBD DW.
